# Neue Literatur zu Verschwörungstheorien

**DOI:** 10.1007/s42520-022-00455-6

**Published:** 2022-09-21

**Authors:** Werner Bührer

**Affiliations:** Technische Universität München, München, Deutschland

**Keywords:** Verschwörungstheorien, Gegenwissen, Konspirationskultur, Wahrheit, Conspiracy Theories, Counter-Knowledge, Conspiracy Culture, Truth

## Abstract

Der Aufsatz gibt einen Überblick über neuere Arbeiten zu Verschwörungstheorien. Ausgehend von der in der einschlägigen Forschung geteilten Erkenntnis, dass politische, sozioökonomische und kulturelle Krisen einen günstigen Nährboden für solche Theorien bilden, wird zunächst dargestellt, wie sich die weltweit grassierende COVID-19-Pandemie auf das Verschwörungsdenken auswirkte – und zwar anhand einiger Neuerscheinungen, die dem Genre der „Verschwörungsliteratur“ zuzurechnen sind. Im Anschluss werden Handbücher vorgestellt, welche den *State of the Art* der Forschung repräsentieren, sowie Arbeiten, die sich mit Verschwörungserzählungen in der Alltags- und Populärkultur beschäftigen. Danach wird die Rolle von Verschwörungstheorien in verschiedenen politisch extremen Milieus beleuchtet: „Reichsbürger“, Rechtsextremisten und -populisten sowie „Querdenker“. Es folgen eine geschichtswissenschaftliche Studie und ethnografisch-kulturwissenschaftliche Arbeiten, die dafür plädieren, sich einer eigene Überzeugungen infrage stellenden Wirklichkeit zu öffnen. Den Abschluss bilden einige Ratschläge zum Umgang mit entsprechenden Erzählungen und eine kurze Erörterung der Frage, ob man angesichts der Relativierung des Wahrheitsbegriffs durch Produzenten und Multiplikatoren von Verschwörungstheorien besser auf diesen Begriff verzichten sollte. Alles in allem lässt sich konstatieren, dass bislang nur wenige Arbeiten zum Thema auf eigenen empirischen Erhebungen etwa zum Selbstverständnis, zur Motivation oder zum soziodemografischen Hintergrund der Anhänger_innen von Verschwörungstheorien basieren.

## Abstract (en)

The essay provides an overview of recent work on conspiracy theories. Books that can be assigned to the genre of conspiracy literature are reviewed, but above all critical works, for example on certain milieus in which conspiracy theories are widespread, on the role of conspiracy thinking in everyday life or on ethnographic and cultural studies approaches. All in all, it can be stated that so far only a few works on the subject are based on their own empirical surveys, for example on the self-image, motivation, or socio-demographic background of the supporters of conspiracy theories.

Verschwörungstheorien handeln von eingebildeten Verschwörungen. Was auf den ersten Blick wie ein offenkundiges Unterscheidungsmerkmal zwischen realen und fiktiven Verschwörungen aussieht, entpuppt sich bei näherem Hinsehen allerdings als weniger eindeutig. Wer entscheidet, ob ein Verdacht oder eine Wahrnehmung tatsächlich begründet sind – oder als pure Einbildung eingestuft werden müssen? Immerhin konnten in der Geschichte seit Lucius Sergius Catilina um 60 v. Chr. zahlreiche *reale* Verschwörungen aufgedeckt werden, wie der Berliner Historiker Wolfgang Wippermann zeigt^1^: Er unterscheidet zwischen religiösen und säkularen Verschwörungsorganisationen. Darunter versteht er „geheime Zusammenschlüsse mehrerer Personen zu einem religiös oder ideologisch motivierten politischen Zweck, den man sowohl positiv wie negativ bewerten“ (S. 23) könne. Die Beispiele, die er anführt, umfassen jüdische wie islamische, christliche wie liberale, (anti-)kommunistische wie (anti-)faschistische Verschwörungen; gelegentlich deutet Wippermann sogar „Kontinuitätslinien“ an – etwa zwischen den jüdischen Zeloten und den islamischen Assassinen. Mit anderen Worten, oft lässt sich erst *ex post*, also nach Klärung aller Umstände, zweifelsfrei feststellen, ob ein reales oder fiktives ‚Komplott‘ existierte. Manche Forscher plädieren deshalb dafür, das ‚Wahrheitskriterium‘ grundsätzlich außer Acht zu lassen und Verschwörungstheorien „dem Bereich heterodoxen (also allgemein nicht anerkannten) Wissens“^2^ zuzuordnen.

Konsens herrscht in der einschlägigen Forschung indes darüber, dass politische, sozioökonomische und kulturelle Krisen einen guten Nährboden für Verschwörungserzählungen bilden. Insofern verwundert es nicht, dass die seit Anfang 2020 weltweit grassierende COVID-19-Pandemie nicht nur ‚neue‘ Erzählungen und ‚Theorien‘ hervorgebracht, sondern auch die kritische Auseinandersetzung mit Verschwörungstheoretiker_innen und Verschwörungsdenken angespornt hat. Einige der so entstandenen Publikationen werden im Folgenden vorgestellt: überwiegend politik- und sozialwissenschaftliche Studien, teilweise ‚Ratgeberliteratur‘ mit Vorschlägen zum Umgang mit Verschwörungsgläubigen, einzelne zeithistorische Arbeiten. Und nur wenige dieser Bücher, das sei vorweg erwähnt, basieren auf eigenen empirischen Erhebungen etwa zum Selbstverständnis, zur Motivation oder zum soziodemografischen Hintergrund der Anhänger_innen von Verschwörungserzählungen. Zunächst aber werden in exemplarischer Absicht fünf Bücher vorgestellt, die dem Genre „Verschwörungsliteratur“ zuzurechnen sind und in unterschiedlicher Weise die Corona-Pandemie mit älteren und umfassenderen Verschwörungstheorien beziehungsweise charakteristischen Elementen solcher Theorien verbinden.^3^

Zuvor noch eine kurze Bemerkung zur Verwendung des Begriffs Verschwörungs*theorie*: Bekanntlich lehnen einige Forscher_innen den Begriff ab, weil es sich dabei nicht um eine Theorie im wissenschaftlichen Sinn handle.^4^ Andere, wie Michael Butter, Karl Hepfer oder Andreas Anton und Alan Schink, plädieren für den Gebrauch, weil, so Butter, durchaus „Gemeinsamkeiten“ mit wissenschaftlichen Theorien vorhanden seien.^5^ Deswegen und angesichts der schlichten Tatsache, dass die meisten Autor_innen den Begriff nach wie vor benutzen, wird er auch hier verwendet – nicht ohne, aus stilistischen Gründen oder wenn die zitierten Studien dies nahelegen, gelegentlich alternative Begriffe zu gebrauchen.

## Die Corona-Pandemie als Impuls für ‚neue‘ Verschwörungstheorien

Paul Schreyer, ‚freier‘ Journalist und Mitherausgeber des Magazins „Multipolar“, das – nach eigenem Verständnis – für „multiperspektivischen Journalismus“ steht und als Organ der ‚Gegenöffentlichkeit‘ gelten kann, geht in seinem neuen Buch der Frage nach, wie „ein Virus die Welt verändern konnte“, so der Untertitel^6^. Der Politikwissenschaftler Markus Linden stuft Schreyer als „vergleichsweise geschickte[n] Verschwörungstheoretiker“ ein^7^, und tatsächlich verzichtet Schreyer auf allzu apodiktische Urteile. Dazu zwei Beispiele: In einem Überblick über die Anfänge der Berichterstattung zur Corona-Krise präsentiert Schreyer zunächst Anhaltspunkte dafür, dass das Johns Hopkins Center for Health Security, das World Economic Forum (WEF) und die Gates Foundation in einer präzise abgestimmten Aktion Mitte Januar 2020 vor einer schweren Pandemie gewarnt und damit das „schlagartig und unvermittelt“ am 20. Januar, einen Tag vor Beginn der Tagung des Weltwirtschaftsforums, einsetzende Medieninteresse ausgelöst hätten. Daraus zieht er die Schlussfolgerung: „Alles war vorbereitet“, nach dem Ende des Davoser Treffens am 24. Januar „entfaltete sich die Krise fast wie automatisch. Die große Pandemie-Maschine, jahrelang konstruiert, geprobt und für den Ernstfall vorbereitet, lief nun.“ Zwar relativiert er direkt im Anschluss diese Beobachtung – sie unterstelle „noch keine Planung oder bewusste Herbeiführung der Pandemie“, der Ablauf lasse sich „auch ganz harmlos erklären“ (S. 123) –, um sich dann aber doch wieder anderen „denkbaren“, weniger harmlosen Erklärungen zuzuwenden, nämlich, dass das Virus als „Biowaffe“ entwickelt oder als „ähnlich wirkende, aber weitaus tödlichere Biowaffe“ im „Schatten eines natürlich aufgetretenen Virus“ (S. 124) eingesetzt worden sein könnte. Das WEF, die Gates-Stiftung und andere transnationale Akteure spielten bei der Steuerung der öffentlichen Wahrnehmung des Corona-Problems, so legt Schreyer nahe, jedenfalls eine zentrale Rolle. Zweites Beispiel: Schreyer räumt immerhin ein, dass COVID-19 eine „für viele Menschen sehr gefährliche Erkrankung“ sei, die „zu großem Leid geführt“ habe und nicht „verharmlost oder kleingeredet werden“ solle (S. 7), um dann genau Letzteres zu tun, indem er behauptet, manche in der Öffentlichkeit verbreiteten „Gewissheiten zum Virus und zur Pandemie“ hielten „einer näheren Überprüfung allerdings nicht stand“ (S. 8).

Mit diesen vermeintlichen Unklarheiten beschäftigt sich das Buch. Im Mittelpunkt steht die Vorgeschichte der Corona-Krise: Schreyer schildert insbesondere Planspiele und Übungen zum Umgang mit Pandemien, wie sie beispielsweise unter der Führung des Center for Health Security durchgeführt wurden, und erwähnt den Einfluss „extrem reicher Menschen“ – „Bloomberg, Rockefeller und Hopkins“ (S. 52) – beispielsweise auf Forschungen zur* biosecurity*. Seit 2005, so Schreyers Beobachtung, begann sich „der Fokus der *bio security-*Gemeinde immer mehr von Terroranschlägen auf Pandemien zu verschieben“ (S. 77). Ein „wichtiger Treiber für die öffentliche Aufmerksamkeit rund um das Thema Pandemien“ seien die „wirtschaftlichen Interessen der Pharmabranche“ geblieben (S. 80). Für die „immer hysterischer werdende Atmosphäre“ (S. 129) macht Schreyer vor allem das mediale „Corona-Dauerfeuer“, die schwindende Fähigkeit der Öffentlichkeit, „ruhig und reflektiert abzuwägen“ (S. 126), und den wachsenden Druck der WHO auf die Regierungen, für eine ausreichende Zahl an Krankenhausbetten, Isolierstationen und Beatmungsgeräten zu sorgen, sowie Behörden wie das Robert-Koch-Institut (RKI) verantwortlich. Nach ein „paar Wochen überhitzter, uniformer“ Medienberichterstattung schien sich Mitte März 2020 die „düstere Utopie einer verängstigten, unfreien Gesellschaft […] realisiert zu haben“ (S. 152). Ein wichtiges Überwachungsinstrument sieht Schreyer übrigens in der Corona-Warn-App; sie sei eines der Systeme, deren „Missbrauchspotenzial mit der Anzahl der Teilnehmer in schwindelerregende Höhen“ wachse (S. 156). Angesichts der mittlerweile mit dieser App gesammelten Erfahrungen mutet diese Befürchtung einigermaßen grotesk an.

Das Schreckensszenario, das Schreyer ungeachtet solcher wenig stichhaltiger ‚Beweise‘ entwirft, sieht so aus: „Spezielle Technologien und Programme, vorangetrieben von einigen Oligarchen, sollen für alle Menschen auf der Welt bindend werden – ohne jede demokratische Debatte“ (S. 11). Die „populäre Annahme, einige Superreiche würden sich zu neuen Weltherrschern machen“, sei „naheliegend“ (S. 12), wenngleich auch sie anscheinend nur Opfer einer Ideologie automatisierter Effizienz seien. Der bei Verschwörungstheoretikern beliebte Begriff der „Neuen Weltordnung“ taucht bei Schreyer in diesem Zusammenhang zwar nicht explizit auf – doch was sonst könnte mit den eben zitierten Mutmaßungen gemeint sein? Und überhaupt sei das „verschwörungstheoretische Denken […] neugieriger, misstrauischer“, vermute „Täuschungen, gerade auch vonseiten der Autoritäten“ (S. 31), und entwickle sich in einer Situation, die geprägt sei durch die „harmonische Erzählung der großen Eintracht von oben und unten“, zu einem „Mittel geistiger Notwehr“ (S. 33).

Auch Daniel Pinchbeck, laut Klappentext Philosoph, Futurist, Bestsellerautor und Herausgeber des „führenden Internetmagazins für spirituelle Transformation“, ist kein ‚lupenreiner‘ Verschwörungstheoretiker. So distanziert er sich beispielsweise vom QAnon-Verschwörungsnarrativ, nach dem die „herrschende Technokratie-Elite eine satanistische Verbindung von Pädophilen“ (S. 23) sei, oder von der Behauptung, dass Bill Gates ein „Superschurke“ sei, „der sich bewusst dazu verschworen hat, Zwangsimpfungen und Biochips als Instrumente zu benutzen, um die Weltherrschaft zu erlangen und eine Entvölkerung auszulösen“ (S. 77). Gleichzeitig kann er dem QAnon-Narrativ auch positive Aspekte abgewinnen, spiegele es doch eine „intensive, instinktmäßige Abneigung“ einer wachsenden Zahl von Menschen gegen das „Voranschreiten einer konzerngesteuerten Globalisierung“ und gegen den „technokratischen Neoliberalismus“ wider (S. 65), und Gates interessiere sich „vermutlich“ durchaus für „implantierbare Biosensoren“ (S. 93). In seinem Buch^8^, das sich vor allem mit Entwicklungen in den Vereinigten Staaten von Amerika befasst, findet man zahlreiche in Frageform gekleidete Vermutungen etwa über skrupellose Topmanager pharmazeutischer Unternehmen, die „einfach nicht an seriöser Erforschung und Erprobung von Impfstoffen interessiert“ seien, weil sie „genau wissen, dass das sprunghafte Ansteigen chronischer Krankheiten wie Autismus, Asthma und ADHS zu ihrem Vorteil ist“ (S. 87). Hinter dem von ihm diagnostizierten „Zerfall des gesellschaftlichen Zusammenhalts“ (S. 70) vermutet Pinchbeck „neoliberale Technokraten und liberale Plutokraten“ (S. 58), namentlich die „berüchtigte Trilaterale Kommission“ (S. 59), eine Denkfabrik, welche der Politikberater und Politikwissenschaftler Zbigniew Brzezinski und der Bankier David Rockefeller im Jahr 1973 gründeten. Bekanntlich ranken sich um die Trilaterale Kommission und Rockefeller zahlreiche Verschwörungstheorien. Wie Schreyer und der Philosoph Giorgio Agamben ist auch Pinchbeck überzeugt, dass zurzeit die COVID-19-Pandemie – und „in Zukunft vielleicht […] ein anderes Virus“ – dazu missbraucht werde, um unter der Parole „Biosicherheit“ ein Herrschaftssystem zu etablieren, das, so zitiert er den italienischen Philosophen, den „endgültigen Stillstand aller politischen Aktivitäten und gesellschaftlichen Beziehungen als höchste Form von Bürgerbeteiligung“ verkläre (S. 96). Hinsichtlich des Ursprungs des Coronavirus vertritt Pinchbeck die ‚Laborthese‘: Die anfängliche Freisetzung sei zufällig passiert, weil die Sicherheitsvorkehrungen am Virologischen Institut von Wuhan „vielleicht mit Absicht“ unglaublich „nachlässig“ gewesen seien; nach der Freisetzung habe die chinesische Führung – „Psychopaten ohne jegliche Empathie für menschliches Leid“ – jedoch entschieden, „dieses Ereignis als Echtzeit-Gelegenheit für sich zu nutzen“ und zu beobachten, wie die internationale Gemeinschaft reagieren und wie hoch der Schaden sein würde (S. 52 f.).

Während Schreyer und Pinchbeck zumindest Alternativen zu ihren verschwörungstheoretischen Deutungen der Corona-Pandemie erwähnen, ist das bei drei anderen Autoren beziehungsweise Büchern nicht der Fall. Flo Osrainik, ‚kritischer‘ Journalist, präsentiert seine Sicht der Vorgeschichte und Hintergründe der Pandemie anders als Schreyer in einem durchgängig höhnischen, verächtlichmachenden Ton^9^, insbesondere dann, wenn es um die „um Konformität bemühten“ (S. 301) Mainstream-Medien, die Bundesregierung oder das RKI geht. Einschlägige Expertise als Epidemiologe oder Virologe kann er nicht vorweisen – ein Punkt, der Erwähnung verdient, weil er seinerseits dem Virologen Christian Drosten oder dem RKI-Chef Lothar Wieler diese Expertise abspricht. Osrainiks Buch, unterteilt in „Hintergründe“, „Vordergründe“ und „Abgründe“, erzählt eine ziemlich ‚unterkomplexe‘ Geschichte mit den üblichen ‚Schurken‘ und ‚Drahtziehern‘ wie dem Johns Hopkins Center for Health Security, der Johns Hopkins Bloomberg School of Public Health, dem WEF, der Bill & Melinda Gates Foundation, der Rockefeller Foundation, dem Global Business Network, der WHO, Klaus Schwab vom WEF und Bundeskanzlerin Angela Merkel (um nur einige zu nennen) auf der einen und zahlreichen angeblich um Freiheit, Demokratie und Grundrechte besorgten Journalisten wie Schreyer und Boris Reitschuster, der im März 2022 aus formalen Gründen von der Bundespressekonferenz ausgeschlossen wurde^10^, Anwälten wie Robert Kennedy Jr., prominenter Impfgegner aus den USA, und der Gruppe „Anwälte für Aufklärung“, ‚geläuterten‘ ehemaligen Politikern wie Albrecht Müller, von 1973 bis 1982 Leiter der Planungsabteilung im Bundeskanzleramt und mittlerweile Herausgeber der dem verschwörungstheoretischen Milieu zuzurechnenden Plattform „NachDenkSeiten“ sowie Ärzten wie Wolfgang Wodarg und Sucharit Bhakdi auf der anderen Seite.

Nun kann man gewiss darüber streiten, ob die im Zuge der Pandemiebekämpfung ergriffenen Maßnahmen alle verhältnismäßig, angemessen und sinnvoll waren. Etwas ganz anderes ist es, die ‚offizielle‘ Pandemiepolitik grundsätzlich als seit Langem geplanten, großangelegten Angriff der Bundesregierung auf verfassungsrechtlich verbriefte Grundrechte und die Demokratie zu diffamieren oder die Vorgänge um den Angriff auf das Reichstagsgebäude am 29. August 2020 herunterzuspielen oder als Inszenierung der Sicherheitsbehörden darzustellen (S. 416). Diesen wahrlich gravierenden Vorwurf stützt Osrainik auf die „Bundesarbeitsgemeinschaft kritischer Polizistinnen und Polizisten“ – ohne zu erwähnen, dass der Sprecher dieser Arbeitsgemeinschaft, Thomas Wüppesahl, bei mehreren Demonstrationen der „Querdenker“ auftrat.^11^ Völlig haltlos ist auch der Begriff „Gleichschaltung“, der dem bereits erwähnten Albrecht Müller im Zusammenhang mit der Berichterstattung über die „Corona-Demonstrationen“ einfällt (S. 424), oder Osrainiks Behauptung, dass „ja schon Spezialeinheiten zur Terrorbekämpfung gegen Impf-Gegner im Einsatz“ seien (S. 425). Und in diesem Stil geht es weiter, gestützt auf Belege aus „Russia Today Deutsch“, „Rubikon“, „NachDenkSeiten“, „Multipolar“ und anderen einschlägigen Internetquellen, deren Verlässlichkeit schlicht vorausgesetzt wird: Die „umstrittenen PCR-Tests“ werden mit dem „unbeirrt[en] Einzug“ eines „globale[n] Faschismus“ in Zusammenhang gebracht (S. 474), „Widerstand und Meinungsfreiheit“ seien bis auf unbestimmte Zeit verboten (S. 475), die „Normopathie, eine zwanghafte Form der Anpassung an vermeintlich vorherrschende und normgerechte Verhaltensweisen und Regelwerke“ sei „weit ansteckender“ als das Virus (S. 476 f.), und mit Schreyer fragt sich Osrainik, „ob die Coronakrise seit Januar 2020 nicht ein globales Ablenkungsmanöver“ sei, „mit dessen Hilfe die Manager des komplexen internationalen Finanzsystems Zeit gewinnen zur Absicherung ihrer Macht und Kontrolle“ (S. 491). Kurzum, wie der geistesverwandte Publizist Ullrich Mies im Geleitwort behauptet, arbeiteten die „transhumanistischen Psycho- und Soziopathen des *World Economic Forum* unter Klaus Schwab [Hervorh. im Orig.]“ hinter den Kulissen längst „im Schulterschluss mit Regierungen, Geheimdiensten und Militärs gegen die Völker, um deren Willen zu brechen“ (S. 20). Die Corona-Pandemie diene dabei lediglich als Vorwand für den seit Langem geplanten „Great Reset“.

So lautet auch der Titel des Buchs des „Autorenkollektivs Dr. C. E. Nyder“.^12^ Wer sich hinter diesem Pseudonym verbirgt, erfahren die Leser_innen nicht. Die „Namenlosigkeit“ der „einzelnen Denkerinnen und Denker“ soll laut Klappentext sicherstellen, dass „Einsichten, Argumente und Analysen für sich selbst und unabhängig von der Person, die sie vorbringt, stehen können“. Veröffentlicht wurde das Buch im Kopp Verlag, der sich unter anderem auf ‚Enthüllungsliteratur‘ und revisionistische Geschichtsdarstellungen spezialisiert hat und mit einigen Büchern aus dem Verlagsprogramm nicht unwesentlich zur Delegitimierung der Demokratie beiträgt. Das „Autorenkollektiv“ zeichnet die Entwicklung seit dem Fall der Berliner Mauer bis ins Jahr 2020 mit dem Schwerpunkt auf Deutschland nach. Im ersten Teil werden der 11. September 2001, der Irakkrieg, die 2008 beginnende Finanz- und Eurokrise, die Energiewende nach dem Reaktorunfall in Fukushima und die Flüchtlingskrise des Jahres 2015 behandelt. Im zweiten Teil, „Vorarbeiten für den ‚Great Reset‘“ überschrieben, wechselt die Darstellung in den ‚offenen Modus‘, das heißt, der Fokus springt ziemlich unvermittelt zwischen verschiedenen Themenkomplexen hin und her: von der „Zerstörung des Nationalstaats“ über „Fridays for Future“, den „Migrationspakt“ der Vereinten Nationen, den Anschlag am Berliner Breitscheidplatz 2016, der die „ungeheuren Ausmaße der moralischen Verwahrlosung des Merkel-Staates offengelegt“ habe (S. 273), bis zur Klimapolitik mitsamt „Green New Deal“ und dem Infektionsschutzgesetz vom 18. November 2020, das als neues „Ermächtigungsgesetz“ geeignet sei, „die letzten Reste von Demokratie in Deutschland endgültig zu beseitigen und eine Gesundheitsdiktatur zu errichten“ (S. 332). Die Rahmenerzählung für all diese einzelnen Ereignisse und Entwicklungen lautet: Spätestens seit der Finanzkrise 2008 vertiefe sich der „Graben zwischen den herrschenden Eliten und der Bevölkerung“, wende sich „die Berliner Republik weiter vom demokratischen Rechtsstaat ab“. Es entstehe der Eindruck, dass es sich „beim Merkel-Staat nicht zuletzt um den Vorreiter und das Versuchslabor für den Great Reset gemäß den Wünschen der Hohepriester der Kalifornischen Ideologie“ handle (S. 343). Die „sogenannte Willkommenskultur“ habe die „Gräben, die die deutsche Gesellschaft davor schon durchzogen“ hätten, „endgültig in Fronten verwandelt“, Corona habe „die Situation noch einmal verschärft“ (S. 345). Wie sähe diese ‚Neue Weltordnung‘ aus? „Ginge es nach den Transhumanisten, würde aller Wahrscheinlichkeit nach am Ende eine global agierende, genetisch oder mechanisch-digital optimierte Herrenschicht, der neue Weltadel, die Macht innehaben. Der übergroße Rest der Menschheit würde, nicht optimiert, ein Sklavendasein fristen […]“ (S. 347). Deutschland und Europa stünden an einem „schicksalhaften Scheideweg“: Jede und jeder habe die „Wahl zwischen einer Existenz als vereinzeltem, seelenlosen Konsumsklaven inmitten einer kalten Dystopie“ – oder einem „freien, selbstbestimmten Leben innerhalb einer Gemeinschaft, die sich ihrer Identität fraglos aus sich selbst heraus bewusst“ sei (S. 351).

Im letzten der fünf Bücher, welche die COVID-19-Pandemie mit älteren Verschwörungserzählungen verknüpfen, wird dies gleich im Titel deutlich: „Durch Corona in die Neue Weltordnung“.^13^ Peter Orzechowski, Autor, Journalist und von 1995 bis 2013 Dozent an der Akademie der Bayerischen Presse in München, wiederholt die bekannten Behauptungen: eine global operierende Elite nutze das COVID-19-Virus als Vorwand, um die „Grundbausteine jeder Gesellschaft zu zerstören: die Familie, das Vertrauen in Recht und Gesetz und vor allem unsere Freiheit“, so der Klappentext. Wer wissen wolle, wie „unser zukünftiges Schicksal aussieht“, der müsse „Leute wie Buffet, Rockefeller, Gates und Soros studieren“. Denn sie seien es, die „entscheiden, obwohl sie nie gewählt wurden“ (S. 13). Der „Masterplan“ der „großen Entscheider“ (S. 19), basierend auf dem „Mittel des Chaos“ durch „unkontrollierte Zuwanderung“ (S. 22) und der „Propagierung absurder ‚Wertvorstellungen‘ wie Genderismus“ (S. 23) sehe unter anderem die Abschaffung der Nationalstaaten, eine „dauerhafte Überwachung“ (S. 35) und eine „weltweite Zwangsimpfung“ (S. 36) vor. Auch die Gleichsetzung des Infektionsschutzgesetzes vom November 2020 mit Adolf Hitlers Ermächtigungsgesetz, der „Transhumanismus“, also die „Züchtung des neuen Menschen“ (S. 78), die verschiedenen Pandemie-Übungen oder der „Bevölkerungsaustausch“ (S. 129) fehlen in der Auflistung nicht. Und selbstverständlich sind alle diese Schritte „von langer Hand geplant“ (S. 28). Zumindest in Deutschland, so Orzechowskis Fazit, habe es „nie eine Pandemie gegeben, nicht einmal eine Epidemie“; die Ausrufung der Pandemie habe vielmehr „ganz andere Ziele verfolgt“, nämlich „flächendeckende Überwachung und Kontrolle, Zwangsimpfung, ein kompletter Neustart der Wirtschaft und als Endziel eine Neue Weltordnung mit einer von den globalen Konzernen gesteuerten Weltregierung“ (S. 194). Russland spielt in dieser Erzählung übrigens die Rolle des Opfers. Russland habe „die Ukraine nie attackiert“ und „bis dato keinerlei ‚imperialistische Ambitionen‘ gezeigt“; Russland untergrabe „auch nicht ‚westliche Staaten‘“ (S. 163) – eine Ansicht, die vor dem Hintergrund der russischen Annexion der Krim und der Intervention in der Ostukraine seit 2014 auch schon vor dem Überfall auf die Ukraine am 24. Februar 2022 definitiv falsch war.

Allen diesen Büchern ist gemeinsam, dass sie sich überwiegend auf Internetquellen stützen, die Glaubwürdigkeit der ‚eigenen‘ Belege oder Gewährsleute nicht infrage stellen, im Grunde genommen dieselbe Geschichte von ‚Drahtziehern‘ im Hintergrund und einem ‚geheimen‘ Plan zur Errichtung der ‚Neuen Weltordnung‘ erzählen, andere Sichtweisen und Erklärungen ignorieren oder diskreditieren, oft einen höhnischen Ton anschlagen sowie Politiker_innen, Expert_innen und Wissenschaftler_innen, die gegenteiliger Auffassung sind, diffamieren. Die Autoren konstruieren das Szenario einer durch Corona zugespitzten Krise, eines unmittelbar bevorstehenden ‚Entscheidungskampfes‘; die Angst der ‚Mächtigen‘ vor „unserem Aufbegehren“ sei „spürbar – vergleichbar mit der Nervosität der DDR-Regierung in den letzten Tagen ihres Wirkens“ (Orzechowski, S. 194). Gleichzeitig fordern sie für sich Gehör und Respekt und den Verzicht auf stigmatisierende Etikettierungen – Verhaltensweisen, die sie selbst gegenüber ihren Kritikern vermissen lassen. Um diese Kritik an Verschwörungstheorien und am Verschwörungsdenken soll es im Folgenden gehen.

## Verschwörungstheorien: *State of the Art*

Als Einstieg in die Materie eignen sich Handbücher besonders gut. Zwei Neuerscheinungen dieses Genres mit ganz unterschiedlichem Zugriff liegen vor. Der von dem bestens ausgewiesenen Innsbrucker Historiker Helmut Reinalter^14^ herausgegebene Band präsentiert, alphabetisch geordnet, die „wichtigsten Sachbegriffe“ sowie bedeutende „Vertreter von Verschwörungstheorien seit dem 17. und 18. Jahrhundert bis zur Gegenwart“ (S. 11). Sacheinträge finden sich beispielsweise zu den Themenkomplexen Antisemitismus, Bilderberg-Konferenzen, Dolchstoßlegende, „esoterische Ufologie“, Freimaurer, Germanenorden, Illuminaten, Jesuiten, Kennedy-Attentat, Mondlandung, Okkultismus und Obskurantismus, „Protokolle der Weisen von Zion“, Rechtextremismus, Sündenböcke, Teufel, Thule-Gesellschaft, dem ‚11. September 2001‘ sowie Verschwörungstheorien im Internet. Allein vier Beiträge erörtern begrifflich-typologische Fragen und die Unterschiede etwa zwischen einer Verschwörungshypothese, -ideologie oder -theorie und einem Verschwörungsmythos, ohne allerdings wirklich zu überzeugen. Die Einträge informieren meist über die verschwörungstheoretischen Deutungen eines Ereignisses, die Fakten und wichtige Akteure. So erfahren die Leser_innen beispielsweise über die „Bilderberger-Verschwörung“, dass deren Ausgangspunkt eine erstmals von Prinz Bernhard der Niederlande im „Hotel de Bilderberg“ im niederländischen Oosterbeek einberufene Konferenz einflussreicher Personen aus „Politik, Wirtschaft, Militär, Medien, Universitäten, Geheimdiensten und dem Hochadel“ (S. 72) war und die Tagungen „unter Ausschluss der Öffentlichkeit“ stattfanden – ein untrüglicher ‚Beweis‘ für Verschwörungstheoretiker, dass die „Bilderberger“ eine „Weltdiktatur und eine neue Weltordnung“ anstrebten (S. 73).

Als wichtige Personen werden neben anderen Augustin Barruel, Karl Heise, Adolf Hitler, Jan Udo Holey alias Jan van Helsing, Jörg Lanz von Liebenfels, Ludwig Müller von Hausen, Erich Ludendorff, Rudolf von Sebottendorf und Adam Weishaupt porträtiert und eingeordnet. Hier fällt auf, dass – von Holey abgesehen – heutige ‚Erfinder‘ und ‚Multiplikatoren‘ von Verschwörungserzählungen wie David Icke, Alex Jones oder Ken Jebsen fehlen. Hitler wird zum einen als Kopf einer „Verschwörung“ zum politischen Umsturz in Deutschland vorgestellt – was insofern zweifelhaft erscheint, als diese „Verschwörung“ ja gewissermaßen ‚auf offener Bühne‘ im Bürgerbräukeller stattgefunden hatte –, zum anderen als Propagandist einer „jüdisch-freimaurerischen Verschwörung“. Der Schwerpunkt sowohl der sach- als auch der personenbezogenen Beiträge liegt auf antifreimaurerischen und vor allem antisemitischen Verschwörungstheorien im Anschluss an die „Protokolle der Weisen von Zion“, die von Antisemiten in aller Welt bis heute benutzt werden, „um ‚die Juden‘ zu bezichtigen, auf konspirativ-subversive Weise nach der Weltherrschaft zu streben“ (S. 209). Michael Hagemeister handelt die Entstehungsgeschichte und Verbreitung der „Protokolle“ ebenso knapp und präzise ab wie den Prozess in Bern Mitte der 1930er Jahre, der die Echtheit des Pamphlets negierte. Dieser gerichtliche Nachweis der Fälschung tat deren weiterer Verbreitung allerdings keinen Abbruch.

Das Handbuch leistet ungeachtet der Konzentration auf Antisemitismus, Freimaurerei und Illuminaten gute Dienste bei der Widerlegung älterer verschwörungstheoretischer Legenden. Beispielsweise lassen sich die Bestrebungen, der „Ein-Dollar-Note einen Geheimcharakter anzudichten, der die verborgene Herrschaft von Freimaurerei und Illuminatenorden belegen soll“ (S. 260), anhand des betreffenden Artikels leicht konterkarieren. Alle Einträge werden überdies durch hilfreiche Hinweise auf einschlägige Quellen und Spezialliteratur ergänzt. Ein differenzierter Sach- und Personenindex hätte den Nutzen des Buchs allerdings noch erhöht. Ein Fazit des Handbuchs lautet, dass mittlerweile „bei nahezu jedem dramatischen und überraschenden Ereignis“ (Armin Pfahl-Traughber, S. 312) ‚passende‘ Verschwörungserzählungen aufkämen. Eine wirklich gründliche Analyse *neuerer* Entwicklungen auf diesem hochdynamischen Feld findet jedoch nicht statt.

Eine solche umfassende, aktuelle und zugleich höchst differenzierte Auseinandersetzung mit dem Thema leistet das von Michael Butter und Peter Knight edierte Handbuch.^15^ Butter lehrt Amerikanische Literatur und Kulturgeschichte in Tübingen, Knight *American Studies* in Manchester; beide haben bereits wichtige einschlägige Studien veröffentlicht.^16^ Die Herausgeber erheben den Anspruch, mit ihrem Handbuch einen umfassenden Überblick über Verschwörungstheorien als wichtiges soziales, kulturelles und politisches Phänomen der Gegenwart zu geben, kurzum: den *State of the Art* der einschlägigen Forschung abzubilden – ein Anspruch, um es vorwegzunehmen, der vollauf eingelöst wird. Insgesamt 71 Autor_innen präsentieren in 48 Beiträgen „current scholarship along with original research findings“ (S. 7). Gegliedert ist das Buch in fünf Abschnitte: „Definitionen und Forschungsansätze“, „Psychologische Faktoren“, „Gesellschaft und Politik“, „Medien und Vermittlung“ sowie „Geschichten und Regionen“.

Der erste Teil versammelt zehn Aufsätze zu den Forschungsansätzen unterschiedlichster Disziplinen, darunter Geschichts‑, Kultur- und Literaturwissenschaft (Butter und Knight), Philosophie (Juha Räikkä, Juho Ritola), Soziologie (Türkay Salim Nefes, Alejandro Romero-Reche), Politikwissenschaft (Julien Giry, Pranvera Tika) und Sozialpsychologie (Olivier Klein, Kenzo Nera), aber auch ungewöhnlichere Zugriffe aus Bereichen wie Okkulte Kosmologie und Anthropologie (Annika Rabo), Netzwerkanalyse (Estrella Gualda Caballero) und Semiotik (Massimo Leone, Mari-Liis Maddison, Andreas Ventsel). Butter und Knight erinnern in ihrem Beitrag daran, dass die geschichtswissenschaftliche Beschäftigung mit Verschwörungstheorien erst im 20. Jahrhundert begann und deren Status als legitime Wissensform erst seit den 1940er Jahren, nicht zuletzt angestoßen durch Karl Popper, in Zweifel gezogen wurde. Sie zeigen, dass sich seit Richard Hofstadters wegweisendem Aufsatz von 1964 über „The Paranoid Style in American Politics“ die Deutung dieses Phänomens merklich ausdifferenziert und gewandelt hat. In diesem Aufsatz beschreibt Hofstadter „pathologised belief in conspiracy theories as a form of paranoia and claimed, wrongly, that while conspiracy theories had a long history in the U.S.A., they had always ‚been the preferred style only of minority movements‘“ (S. 29). Pathologisierende Interpretationen seien durch ‚neutralere‘ verdrängt worden, die unter Zuhilfenahme kulturgeschichtlicher Zugriffe betonen, wie populär und einflussreich Verschwörungstheorien in ihrem jeweiligen historischen Entstehungszusammenhang gewesen seien. Wichtig ist auch ihr Hinweis auf die Faszination, die von spekulativ-fiktionalen Verschwörungserzählungen ausgeht, verglichen mit wissenschaftlichen Erklärungsversuchen komplexer politischer, ökonomischer oder sozialer Entwicklungen und Sachverhalte. Eine Auseinandersetzung mit solchen Erzählungen über im Geheimen operierende Gruppen (‚Illuminaten‘, ‚Reptiloide‘, ‚Freimaurer‘, ‚Bilderberger‘ *et cetera*) oder Individuen (etwa Bill Gates und George Soros) mit dem Ziel, gigantische Profite einzustreichen und die Weltherrschaft an sich zu reißen, wird dadurch erschwert, dass sie oft auf „the fear of ‚could-be-true‘ but ‚not yet proven‘“ basieren (S. 114), wie Giry und Tika erwähnen.

Die acht Aufsätze des zweiten Teils widmen sich aus unterschiedlichen Blickwinkeln den psychologischen Aspekten des Problems, besonders der Frage, warum manche Menschen an Verschwörungstheorien glauben, andere dagegen nicht. Während drei Beiträge eher persönliche Faktoren in den Blick nehmen, konzentrieren sich drei weitere auf die situativen. Die letzten beiden Artikel untersuchen die gesellschaftlichen Konsequenzen von Verschwörungserzählungen und mögliche Strategien, deren Einfluss zu begrenzen, insbesondere wenn sie Schaden anrichten könnten. Wie lässt sich also die hohe Anziehungskraft auf bestimmte Personen erklären? In ihrem Beitrag über den Zusammenhang zwischen Motivationen, Emotionen und dem Glauben an Verschwörungstheorien konstatieren Karen M. Douglas, Aleksandra Cichoka und Robbie M. Sutton, „conspiracy theories allow people to understand the reasons underlying important events and circumstances in a way that addresses their broader motivational concerns to feel knowledgeable, safe and good about the self“ (S. 181). Der Kenntnisstand über die emotionale Untermauerung sei dagegen noch sehr dürftig. Fest stehe immerhin, dass „experiencing uncertain emotions increased belief in conspiracy theories compared to emotions associated with certainty“; auch „boredom proneness“ begünstige Verschwörungsdenken (S. 186). Jedenfalls, so Péter Krekó im abschließenden Beitrag des zweiten Blocks, sei es einfacher, Verschwörungstheorien zu verbreiten als zu widerlegen, zumal viele gar nicht falsifizierbar seien. Außerdem seien diese Erzählungen zunächst einmal „a legitimate part of the democratic discussion and deserve defence as a form of free speech“, mit anderen Worten, „debunking and regulation might be problematic as a form of political suppression“ (S. 243). Gleichwohl seien Gegenmaßnahmen insbesondere dann unumgänglich, wenn es sich um Theorien handle, die populär, nicht plausibel und schädlich seien.

Im dritten Teil stehen Verschwörungstheorien im Kontext von Gesellschaft und Politik im Fokus. Die insgesamt zwanzig Autorinnen und Autoren fragen nach den Zusammenhängen von Verschwörungsdenken und autoritären Regimen, Populismus, Radikalisierungsprozessen, Antisemitismus und Religion, ferner nach demografischen Merkmalen von Verschwörungstheoretikern und nach der Rolle und dem Erfolgsrezept einzelner ‚Verschwörungsunternehmer‘ wie David Icke und Alex Jones sowie nach Bewegungen wie dem „9/11 Truth Movement“. Einige wichtige Befunde seien erwähnt: Bürger_innen reifer und gut funktionierender Demokratien sind weniger anfällig; die besonders erfolgreichen ‚Stars‘ der Szene wie Icke und Jones haben aus der Verbreitung von Verschwörungstheorien ein lukratives Geschäftsmodell gemacht, indem sie „dark thoughts of a coming totalitarian New World Order with messianic visions of a global awakening into a New Age“ (Jaron Harambam, S. 289) verschmelzen; im Gegensatz zum gängigen Narrativ vom „unwashed, middle-aged white male“ fanden die meisten einschlägigen Studien keine stichhaltigen Belege dafür, „that gender has any impact on belief in conspiracy theories“ (Annika Thiem, S. 292) – die ‚Krise der Männlichkeit‘ stellt gleichwohl einen „key factor fuelling conspiracy theories“ dar (dies., S. 302); es gibt einen eindeutigen Zusammenhang zwischen Populismus und Verschwörungstheorien – „but how or to what extent has not yet been adequately theorised“ (Eiríkur Bergmann/Butter, S. 340); beim Thema Religion muss klar unterschieden werden zwischen Verschwörungstheorien *als* Religion, *über* und *in* Religionen. Auch wenn manche Beiträge fast genauso viele Fragen aufwerfen, wie sie Antworten geben, bündelt das dritte Kapitel den Kenntnisstand auf höchst unterschiedlichen Forschungsfeldern in hervorragender Weise.

Die zehn Aufsätze des vierten Teils handeln, so Stef Aupers, Dana Crǎciun und Andreas Önnerfors in ihrem Vorspann, „in one way or another, with conspiracy theories *as/in* media texts and their historical and multimodal transmission *through* media [Hervorh. im Orig.]“ (S. 390). Das Spektrum reicht von Gerüchten und der mündlichen Überlieferung von Verschwörungstheorien über die Thematisierung von Verschwörung und Paranoia in der amerikanischen Literatur seit den 1960er Jahren – in den Werken etwa von Thomas Pynchon, Don DeLillo, Margaret Atwood, Philip K. Dick und William Gaddis, um nur einige zu nennen – und die visuelle Repräsentation von Verschwörungserzählungen auf Gemälden, Lithografien, in Filmen und Fernsehserien bis zur zentralen, aber ambivalenten Rolle des Internets bei der Verbreitung von Verschwörungstheorien und Desinformation sowie der Frage, was Erstere und ‚Fake News‘ verbindet oder trennt.

Im abschließenden fünften Teil bieten die Autor_innen in elf Artikeln einen Überblick über Verschwörungstheorien in verschiedenen historischen Epochen sowie in unterschiedlichen Ländern und Regionen. Dabei wird erneut deutlich, dass bestimmte Erzählungen, insbesondere antisemitisch aufgeladene, auf eine lange Tradition vom Mittelalter bis in die Gegenwart zurückblicken können. Freimaurer und der Illuminatenorden ergänzten den Kreis der ‚üblichen Verdächtigen‘ seit der Französischen Revolution. Sowohl der Erste als auch der Zweite Weltkrieg und der anschließende Kalte Krieg boten ideale Bedingungen für den Glauben, dass – je nach politischer Zweckmäßigkeit – jüdische, freimaurerische, kommunistische oder westliche Spion_innen, Verräter_innen und Umtriebe für Niederlagen, Katastrophen oder Umsturzversuche verantwortlich seien. Ein neues Phänomen waren die seit den 1970er Jahren an Einfluss gewinnenden „anti-establishment conspiracy theories“. Zunächst auf die ‚Erklärung‘ einzelner Morde, Selbstmorde oder Unfälle – Olof Palme, Uwe Barschel, Prinzessin Diana – zugeschnitten, „those conspiracy theories progressively developed into superconspiracy theories“ (Pascal Girard, S. 578). Sehr informativ sind die Beiträge über Regionen (Balkan, Naher Osten, Südostasien) und Länder (Türkei, Vereinigte Staaten, Venezuela), welche die verbreitete eurozentrische Perspektive überwinden und verdeutlichen, dass es sich keineswegs um ein ‚westliches‘ Phänomen handelt: „indeed, conspiracy theories have become driving forces for creating national political identities, and help governments channel often quite legitimate political, economic and social dissatisfaction onto the supposedly dangerous conspiring ‚other‘“ (Ilya Yakblokov, Girard, Nebojša Blanuša, Rabo, S. 529). Besondere Aktualität hat der Aufsatz über Verschwörungstheorien in Putins Russland erlangt. Zwar spielte die Ukraine und ein angeblicher ‚Völkermord‘, verübt an der russischen Minderheit in der Ostukraine, in den zahlreichen Verschwörungstheorien, die nach dem Zusammenbruch der Sowjetunion entstanden, noch keine nennenswerte Rolle – wohl aber die Behauptung, dieser Zusammenbruch sei das Werk des ‚Westens‘ und der ‚Agenten der Neuen Weltordnung‘ gewesen. Die meisten Stereotype gängiger westlicher Verschwörungserzählungen finden ihren Niederschlag auch in russischen Varianten: „das Komitee der 300“, die Trilaterale Kommission, David Rockefeller, die World Trade Organisation – und auch die vermeintliche Auslöschung der – in diesem Fall russischen – Bevölkerung durch „genetically modified food, the promotion of L.G.B.T. marriages that don’t produce children and through epidemics that would kill 80 percent of the global population“ (Yablokov, S. 592) fehlt nicht. Als Legitimationsquelle für eine autoritäre Politik eignen sich solche Erzählungen allemal.

Es dürfte deutlich geworden sein, dass dieses Handbuch dank der Fülle an empirischen Befunden, unterschiedlichen disziplinären Zugriffen, differenzierten Informationen und Literaturhinweisen sowie Fingerzeigen auf Forschungslücken ein unverzichtbares Kompendium und Hilfsmittel für alle Forscher_innen darstellt, die sich mit Verschwörungstheorien beschäftigen wollen. Komplettiert wird das in jeder Hinsicht überzeugende Werk durch einen ausführlichen Sach- und Personenindex.

Wer sich im Übrigen für die Verbreitung von Verschwörungstheorien besonders in und über Europa interessiert, dem sei ein von Andreas Önnerfors und André Krouwel herausgegebener Band empfohlen, der sich dieser Thematik aus unterschiedlichen und ungewohnten Perspektiven nähert^17^: So fragen, um nur einige der durchweg sehr interessanten Themen zu erwähnen, Krouwel und Jan Willem van Prooijen nach dem Zusammenhang von Euroskeptizismus und Verschwörungsglauben, Eirikur Bergmann analysiert die Verschwörungserzählungen um „Eurabia“, also die Behauptung einer drohenden Eroberung Europas durch den Islam, Armin Langer setzt sich mit dem Vorwurf auseinander, dass George Soros’ Eintreten für eine offene, immigrationsfreundliche Gesellschaft die Ausbreitung des Coronavirus begünstigt habe, und Jakov Bojovic spürt unter der Überschrift „The Brussels conspiracy“ den auf die Europäische Union zielenden Verschwörungstheorien in Kreml-nahen Medien nach. Der Politikwissenschaftler Thomas Milan Konda liefert eine solche differenzierte Analyse für die Vereinigten Staaten von Amerika.^18^ Die Anfänge oder anders gesagt, die „Invention of Conspiracy Theory“, datiert er in die Zeiten der Französischen Revolution und des bayerischen Illuminatenordens. Er folgt der Entwicklung dieser Ideen und dieses Denkens über verschiedene Stationen wie den „arischen Okkultismus“, die Behauptung einer „jüdischen Weltverschwörung“, die Entstehung eines „White Supremacist Conspiracism“ (S. 127) und der Idee einer ‚Neuen Weltordnung‘, die Warnungen vor einer „Gehirnwäsche“ mit dem Ziel der Errichtung einer ‚globalen Diktatur‘ (S. 168), der Ausbreitung des „Denialism“ (S. 202), einer Bewegung, die Front machte gegen die Anerkennung wissenschaftlicher Erkenntnisse bis hin zu den Gruppierungen der „Truther“ und der „Birther“ sowie den rechtsextremistischen Milizen. Sein ernüchterndes Fazit lautet: „Conspiracism enters the mainstream“ (S. 283). Seine Hoffnungen, dem Siegeszug des verschwörungstheoretischen Denkens etwas entgegensetzen und die Demokratie verteidigen zu können, ruhen nicht zuletzt auf der wissenschaftlichen ‚Community‘ und der Zivilgesellschaft. Allzu optimistisch wirkt Konda freilich nicht.

## Verschwörungserzählungen in der Alltags- und Populärkultur

Wenn man überhaupt einen kritischen Einwand gegen das von Butter und Knight edierte Handbuch vorbringen wollte, dann den, dass die Autor_innen Verschwörungserzählungen als zentralem Bestandteil der Alltags- und Populärkultur eher wenig Beachtung schenken. Genau diesem Komplex widmen sich die 24 Beiträge des abwechslungsreichen Bandes, den Brigitte Frizzoni, Dozentin am Institut für Sozialanthropologie und Empirische Kulturwissenschaft der Universität Zürich, herausgegeben hat.^19^ Ihr Interesse gilt den „genreübergreifenden, die Grenzen von Fiktionalität und Faktualität überschreitenden Verschwörungserzählungen mit ihren spezifischen Argumentationsmustern, Plausibilisierungsstrategien und Verweissystemen“ (S. 10).

Im ersten Teil stehen „grundlegende Überlegungen […] aus kulturwissenschaftlich-narratologischer, soziologischer und psychologischer Perspektive“ (S. 11) im Mittelpunkt. Harm-Peer Zimmermann präsentiert seine Überlegungen zu einer „Theorie der Verschwörungstheorie“ und kommt im Anschluss an Niklas Luhmann zu dem Ergebnis, dass es sich dabei um ein „autopoetisches [sic!] System“ handle, das „jede Irritation in ein Instrument zur eigenen Stabilisierung umwandelt, und zwar dermaßen hermetisch abgeriegelt und in sich kreisend, dass jede Kontingenztoleranz ausgeschlossen ist: Selbstreferenzialität in Reinkultur“ (S. 32). Regina F. Bendix erinnert in ihrem Beitrag über „Geheimhaltung: Kulturelle Praxis und narrativer Ausgangspunkt für soziales Misstrauen“ daran, dass „Geheimnisse und Geheimhaltung integraler Bestandteil zwischenmenschlichen Daseins“ seien (S. 36). „Gerade weil ein jeder und eine jede seit der Kindheit mit dem Prinzip Geheimnis vertraut ist, gedeihen Vermutungen, dass Geheimes vorhanden sein könnte – von ungeteiltem Wissen bis zum Vorhandensein untergründiger, umsturzplanender Organisationen“ (S. 44). Sabine Wienker-Piepho geht aus der Perspektive einer „historisch-vergleichenden Erzählforschung“ (S. 49) dem „Elvis-lebt-Narrativ“ nach, Andreas Anton beklagt, dass „Verschwörungstheorien regelmäßig in einen problematisierenden, negativen oder sogar pathologisierenden Zusammenhang“ gestellt würden (S. 64) – Verschwörungstheorien könnten „nicht nur negatives, sondern durchaus auch positives gesellschaftspolitisches Potenzial aufweisen“, indem sie beispielsweise „zur Aufdeckung tatsächlicher Verschwörungen dienen, Betrug oder Machtmissbrauch aufzeigen“ oder „auf ökonomische Manipulationen oder politische Korruption hinweisen“ (S. 71). Ähnlich argumentieren auch Julian Genner und Ina Dietzsch, die im Anschluss an Pierre Bourdieu „‚Verschwörungstheorien‘ als Teil einer Konkurrenz um die ‚richtige Weise, Leben und Welt zu erleben und zu sehen‘“ verstanden wissen wollen (S. 77). Sebastian Dümling befasst sich mit „Verschwörungsbeobachtungen“ in verschiedenen Dramen Friedrich Schillers. Dieses Phänomen erklärt Dümling damit, dass Stücke wie „Maria Stuart“ oder „Wilhelm Tell“ „Institutionengenese und -stabilisierung“ thematisierten – Prozesse, die „Misstrauen gegenüber Institutionen“ begünstigten (S. 89). Philosophische und psychologische Zugänge zum Verständnis von Verschwörungstheorien stehen im Mittelpunkt des Aufsatzes von Bernd Rieken, ehe Anna Jank den ersten Teil des Buches mit einem persönlich gefärbten Bericht über eine psychisch labile Person beschließt, die nach eigenem ‚Erleben‘ „weiter und weiter in die Tiefe einer perfiden Verschwörung gezogen“ (S. 120) wird und schließlich „Selbstmord aus Todesangst“ begeht (S. 123).

Die Autorinnen und Autoren des zweiten Teils loten die Alltagsrelevanz von Verschwörungserzählungen aus. Alice Blum und Michael Urmoneit zeigen, gestützt auf ethnografisch erhobenes Material, welche Rolle Verschwörungsideologien im Alltag „völkischer Siedlerinnen und Siedler“ spielen (S. 128). Marion Näser-Lather diskutiert Verschwörungserzählungen rund um das Thema „Gender Mainstreaming“ und speziell um das Fach „Gender Studies“: ein „zentrales Motiv“ dieser Erzählungen stelle „die angebliche Verschwörung von Feministinnen auf der Weltfrauenkonferenz von Peking“ im Jahr 1995 dar, „als deren Ziele die Förderung der Homosexualität, die Bekämpfung christlicher Werte und der bürgerlichen heterosexuellen Familie und die Abschaffung der Geschlechter imaginiert“ werde (S. 143); Anklänge an die „Protokolle der Weisen von Zion“ sind nicht zu übersehen. Mit „Celebrity Gossip“ am Beispiel der amerikanischen Pop-Künstlerin Taylor Swift beschäftigt sich Fatma Sagir, mit Verschwörungserzählungen im Fußballmilieu – etwa über die mitunter spielentscheidende Rolle von Schiedsrichtern oder den angeblich vom Deutschen Fußballbund und der Deutschen Fußballliga orchestrierten „Bayern-Bonus“ – Christina Niem. Die übrigen Aufsätze des zweiten Teils widmen sich hierzulande eher weniger geläufigen, via Internet und Film allerdings weltweit verbreiteten Verschwörungserzählungen und *contemporary* oder *urban legends* beispielweise über die Verwendung von mit Blattern verseuchten Decken zur Dezimierung von Indianerstämmen (Mirko Uhlig), den „Mothman“ (Janin Pisarek), die „Menstruationslüge“ (Pauline Lörzer) und die speziell in Japan hinter der Zahl 666 vermuteten Geheimnisse (Akemi Kaneshiro-Hauptmann) – übrigens nicht nur dort, gilt diese Zahl doch beispielsweise in verschwörungsgläubigen, rechtsesoterischen oder evangelikalen Kreisen als Chiffre für den Antichrist oder den Teufel. Die Vielfalt der Themen vermittelt einen guten Eindruck von der enormen Bedeutung, welche über die modernen Massenmedien transportierte verschwörungstheoretische Narrative in der heutigen Populärkultur haben – zumal dann, wenn es deren Multiplikatoren gelingt, regionale, traditionelle oder milieuspezifische Motive mit globalen Diskursen zu verbinden.

Die Einzelfallanalysen des dritten Teils konzentrieren sich auf die Ausgestaltung von Verschwörungserzählungen in Romanen, YouTube-Videos, Dokumentarfilmen, TV-Serien und Radiosendungen. Alfred Messerli rekapituliert den vor Schweizer Gerichten in den 1930er Jahren ausgetragenen Streit über die Echtheit der „Protokolle der Weisen von Zion“ und das Urteil, es handle sich um „Schundliteratur“ im „ästhetischen, literarischen, aber nicht im rechtlichen Sinne“ (S. 245). Meret Fehlmann spürt den Zusammenhängen zwischen Geschichten über „antike Astronauten oder Astronautengötter“, über „geheimes, verworfenes Wissen“ und Verschwörungserzählungen nach (S. 257). Iraj Esmaeilpour Ghoochani und Tilman Weinig befassen sich mit einem Roman des iranischen Schriftstellers Iraj Pezeshkzad, der die „Empfänglichkeit“ der iranischen Bevölkerung für eine Verschwörungstheorie, nach der ‚die Engländer‘ hinter allen schädlichen Entwicklungen steckten, verspottet – und „dennoch bei derselben Bevölkerung äußerst populär“ sei (S. 275). Johannes Glaser zeigt anhand von Kanälen wie „NuoViso“ und „KenFM“, dem Webportal eines ehemaligen Journalisten beim Rundfunk Berlin-Brandenburg, der unter dem ‚Künstlernamen‘ Ken Jebsen einschlägige Verschwörungstheorien verbreitet und dessen Portal seit März 2021 vom Berliner Verfassungsschutz als „Verdachtsfall“ eingestuft wird, sowie einzelnen in der deutschen Szene bekannteren ‚Verschwörungsunternehmern‘ wie Oliver Janich und Tilman Knechtel, wie Verschwörungserzählen in einer digitalisierten Welt funktioniert (S. 287). Die *chemtrails*-Erzählungen untersucht Simone Stiefbold am Beispiel einer ‚Expertin‘ für dieses Phänomen, die davon überzeugt ist, dass „wir aus Flugzeugen besprüht werden, weil ‚die da oben‘ die Bevölkerung reduzieren wollen, *mind control* betreiben und Wetterexperimente machen“ (S. 296), und die ihre Erfahrungen in einer Art „Erweckungserzählung“ (S. 300) auf YouTube präsentiert. Mit dem im einschlägigen Milieu besonders einflussreichen und stilprägenden ‚dokumentarischen‘ Film „Zeitgeist“ setzt sich Deborah Wolf auseinander. Der Film konstruiert in typischer Manier aus disparaten Ereignissen eine „zusammenhängende Geschichte“, indem er diese Ereignisse „als Verschwörungsbeobachtungen interpretiert und in die (Welt‑)Verschwörungstheorie integriert“. Dadurch, so Wolf, entstehe ein „hoher Grad an Kohärenz“, Verschwörungserzählungen seien somit „‚kohärenter als die Realität‘“, und genau dies mache ihren „Reiz“ aus (S. 313). Bedauerlicherweise übernimmt Wolf an einer Stelle die falsche Begrifflichkeit des Films, wenn sie von den „Reichsermächtigungsgesetzen“ [sic!] des Nationalsozialismus spricht. Die letzten beiden Aufsätze handeln von der Fernsehserie „24“, in der nach Ansicht Malte Völks immerhin die Aufforderung mitschwinge, „die ganzen Verschwörungserzählungen nicht mit der Realität zu verwechseln“ (S. 332), sowie der zwischen 2002 und 2015 ausgestrahlten spanischen Radiosendung „Milenio 3“, die sich „dem Paranormalen und Übernatürlichen“ (S. 16) widmete und von Beginn an auch Verschwörungen thematisierte, wie Marina Jaciuk zeigt.

Der Band, der Beiträge vor allem aus den Fächern Kulturanthroplogie und Germanistik enthält, lenkt den Blick von der ‚großen‘ politischen Bühne auf den Bereich der Alltags- und Populärkultur. Selbst wer ‚großen‘ Erzählungen à la „Neue Weltordnung“ oder „Großer Austausch“ distanziert oder ablehnend gegenübersteht, wird sich möglicherweise hin und wieder dabei ertappen, dass ‚alltägliche‘ Verschwörungserzählungen in populären Filmen, Romanen oder Comics eine gewisse Faszination ausüben können. Insofern ist es enorm wichtig, ihnen die gebührende Aufmerksamkeit zu schenken, so wie Frizzoni und ihre Mitstreiter_innen es getan haben.

## Verschwörungserzählungen, „Reichsbürger“, Rechtsextremismus und Populismus

Verschwörungstheorien und Verschwörungsdenken spielen bekanntlich auch in verfassungsfeindlichen, rechtsextremistischen und (rechts-)populistischen Bewegungen, Organisationen und Milieus eine maßgebliche Rolle. Dementsprechend greifen mehrere neuere Studien diese Problematik auf.

Die „Reichsbürger“ galten lange Zeit als eine Bewegung, deren Anhänger bestenfalls als kauzig, schlimmstenfalls als lächerlich oder verrückt wahrgenommen wurden. Seit ein „Reichsbürger“ im Herbst 2016 auf ‚seinem‘ Grundstück einen Polizisten erschoss und wegen Mordes zu lebenslanger Haft verurteilt wurde, hat sich diese Wahrnehmung grundlegend gewandelt. Inzwischen wird die Szene als ernste und unbedingt ernstzunehmende Bedrohung für Demokratie und Rechtsstaat verstanden. In ihrem schmalen Band beleuchten Christoph und Sophie Schönberger, beide lehren Öffentliches Recht, zusammen mit ihren neun Autor_innen verschiedene Aspekte dieses Phänomens.^20^ Den Kern der Ideologie sehen die beiden in einer „fundamentalen Delegitimierung der bestehenden Staats- und Rechtsordnung“, auf welches „vergangene Reich“ sich die „Reichsbürger“ genau beziehen, bleibe „häufig im Vagen“; in deren Selbstverständnis trage „der Bezug auf das Deutsche Reich Züge einer sektenhaften Wahrheit, die bekannt und geglaubt werden muss, ohne dass eine Widerlegung denkbar wäre“. Zu dieser Bewegung fänden „nicht selten Menschen, deren Zugehörigkeit zur Gesellschaft prekär“ sei, „weil sie sich in persönlichen, familiären oder beruflichen Krisen“ befänden (S. 20). Hier interessieren jedoch nicht die soziologischen oder verfassungsrechtlichen Aspekte der Szene, sondern ihre ideologische ‚Schnittmenge‘ mit Verschwörungstheorien. Damit setzt sich der Psychologe Marius Hans Raab auseinander. Wenn, so sein Argument, „fast alle der über 82 Millionen Deutschen der Meinung sind, dass die Bundesrepublik ein legitimer Staat ist, dann muss die vermeintliche Wahrheit […] sehr gut verborgen sein“. Nur die „mächtigsten Politiker, Wirtschaftsführer, Richter und Professoren hätten wohl die Macht, uns einen rechtmäßig verfassten Staat vorzugaukeln. Die angenommene Verschwörung ist damit groß, mächtig, und existiert seit mindestens 1945“ (S. 111). Inwiefern ein solches alternatives Weltbild gefährlich werden oder aber „aus der Sicht der Betroffenen“ sogar „eine positive und sinnstiftende Funktion“ haben könnte (S. 121), bedarf laut Raab noch genauer Erforschung. Eine umfangreiche, in formaler Hinsicht allerdings gewöhnungsbedürftige Studie von Gerhard Schumacher^21^ liefert zwar reichlich Material zur Auseinandersetzung mit den Behauptungen und Standpunkten der „Reichsbürger“, geht aber auf das Thema Verschwörungstheorien nur ganz kurz ein (zum Beispiel S. 12) und macht es sich, was das Urteil über die Szene angeht (‚Verrückte‘), doch etwas zu einfach.

Die „Liaison von Rechtsextremismus und sozialen Medien“ untersuchen die Sozialwissenschaftler Maik Fielitz und Holger Marcks.^22^ Sie weisen darauf hin, dass Rechtsextremisten Verschwörungserzählungen als Rahmen für Fake News und Gerüchte nutzen. Solche Erzählungen wirkten in diesem Milieu als identitätsstiftend und ermöglichten es, „auch eklatanteste Widersprüche zwischen den eigenen Behauptungen und der statistisch vermittelbaren Realität, also den Fakten“, aufrechtzuerhalten (S. 108). Beide Phänomene zeichneten sich überdies durch ein „sehr spezielles Mitläufertum“ aus, das der gemeinsamen „Vorliebe […] für das Postfaktische“ und einem gegen das „Establishment“ gerichteten „Rebellengestus“ (S. 148 f.) geschuldet ist. Die extreme Rechte profitiere dabei von weitverzweigten Netzwerken beispielsweise von „rechtsextremen Youtubern und alternativen Nachrichtenformaten, die sich durch Verlinkungen und Empfehlungen wechselseitig“ unterstützten (S. 153). In rechtsextremistischen Kreisen besonders weit verbreitet ist die Verschwörungserzählung vom ‚Großen Austausch‘. Auf diese und andere einschlägige Erzählungen gehen Andreas Anton und Alan Schink in ihrem Überblick über reale und fiktive Verschwörungen kurz ein.^23^ Das Schlagwort fand Verbreitung vor allem durch eine Publikation des französischen Autors Renaud Camus^24^, der in rechtsextremistischen und „identitären“ Kreisen hohes Ansehen genießt, und erhielt durch das Scheitern der Asyl- und Migrationspolitik in der EU angesichts des Anstiegs der Flüchtlingszahlen im Herbst 2015 neue Nahrung. Eine nicht genauer identifizierte „Machtclique“ verfolge „mit allen finanziellen und ideologischen Mitteln“ das Ziel, „das eigene Volk im jeweiligen Staat nach und nach“ auszulöschen (S. 140). Attraktiv für rechte Deutungen, so Anton und Schink, sei überdies die ursprünglich ‚linke‘ Behauptung eines „Deep State“, die auch in der QAnon-Verschwörungsideologie eine wichtige Rolle spielt. Demnach zögen Teile des amerikanischen Staatsapparats, allen voran „Netzwerke der Demokraten um Hillary Clinton“, die in ‚satanistische‘ und ‚pädophile‘ Umtriebe verwickelt seien und Menschenhandel betrieben, „im Hintergrund, aus dem ‚Deep State‘ heraus, die Strippen“ (S. 143). Aber auch in Deutschland gibt es zahlreiche Anhänger der „Deep State“-Verschwörungstheorie. So habe beispielsweise der Attentäter von Hanau entsprechende Versatzstücke in sein Weltbild integriert. In einem Videoclip habe er an seine Zuschauer_innen appelliert, „aufzuwachen und den Kampf aufzunehmen“ (S. 145) – ‚klassische‘ Ausdrücke und Parolen der verschwörungstheoretischen Rhetorik. Zum Schluss des Kapitels erwähnen die beiden Autoren den „Topos von der ‚braunen Verschwörung‘“, also sensationsheischende Berichte über angebliche ‚Super-‘ oder ‚Geheimwaffen‘ und ‚Reichsflugscheiben‘ (S. 152): Der „Topos der ‚Nazi-Verschwörung‘“ schwanke jedenfalls „zwischen Verschwörungen und Spekulationen über sie, Mythisierungen und Fiktionalisierungen“. Rechtsextreme Organisationen und Aktivist_innen glaubten nicht nur an Verschwörungstheorien, sie agierten selbst „in konspirativer Weise“ und regten damit solche Erzählungen an, zumal die „Verbindungen zwischen Rechtsterrorismus und Sicherheitsbehörden sowie das in diesem Zusammenhang oftmals ebenso als konspirativ zu bezeichnende Verhalten staatstragender Institutionen und Akteure“ durch „eine Art ‚strukturelles Tabu‘“ gekennzeichnet seien (S. 153).

Einen anderen Zugriff wählen die Journalistin Heike Kleffner und der Journalist Matthias Meisner.^25^ Gemeinsam mit ihren Autor_innen analysieren sie vor dem Hintergrund der Coronavirus-Pandemie, ob und wie die „Normalisierung von Verschwörungsnarrativen, Wissenschaftsfeindlichkeit, Antisemitismus und Rassismus die Koordinaten politischen Handelns und gesellschaftlichen Zusammenlebens verschieben“ (S. 13). Der Journalist Felix Huesmann lenkt den Blick auf die „ideologischen Fragmente“, welche die aus den USA importierte und mittlerweile in Deutschland ebenfalls recht einflussreiche QAnon-Erzählung „anschlussfähig für Teile der hiesigen extremen Rechten, der Reichsbürgerszene und andere Verschwörungsideologen machen“ (S. 109). Dass Verschwörungserzählungen in extremen Gruppierungen als „Radikalisierungsbeschleuniger“ (S. 117) wirken können, belegen die Psychologin Pia Lamberty und die Sozialwissenschaftlerin Katharina Nocun. ‚Anfällig‘ für Verschwörungstheorien seien „insbesondere Menschen mit einem starken Bedürfnis nach Einzigartigkeit“ (S. 121). Der einschlägig ausgewiesene Historiker Volker Weiß zeigt in seinem Beitrag über die „Neue Rechte in Pandemiezeiten“, wie „Protagonisten der äußersten Rechten“ versuchen, die Proteste gegen die Maßnahmen zur Bekämpfung der Corona-Pandemie zu ‚unterwandern‘, weil sie glauben, dass diese Proteste ein „ähnliches Mobilisierungspotenzial“ in sich bergen wie jene gegen Migration und Flüchtlinge (S. 158 f.). Dem Thema „Neonazis bei den Coronaprotesten“ widmet sich auch der Journalist Konrad Litschko. Er konstatiert, dass es den Neonazis „leicht gemacht“ werde, sich einzureihen, artikulierten die Kundgebungen doch vieles, was zum „rechtsextremen Kanon“ gehöre: „Agitation gegen Regierung und ‚Lügenpresse‘, die Dichotomie von böswilligen Eliten einerseits und einem unterdrückten Volk andererseits, dazu teils antisemitisch aufgeladene Verschwörungserzählungen“ (S. 183).

Warum Populismus und Verschwörungsdenken so gut harmonieren, ist ein wichtiges Thema der Studie von Katrin Götz-Votteler und Simone Hespers.^26^ Sie machen dafür einige „strukturelle Überschneidungen“ verantwortlich, vor allem ein „Wir-gegen-die-Anderen“-Narrativ als „ideologischen Kern beider Phänomene“, einen „Vertrauensverlust gegenüber den Eliten“ sowie die Berufung auf den ‚gesunden Menschenverstand‘, die „Reduktion komplexer Zusammenhänge“ und eine simple, monokausale, „von Fakten losgelöste und emotional aufgeladene Argumentation“ (S. 164). Sowohl Populismus wie Verschwörungstheorien profitierten überdies von „aufmerksamkeitsökonomischen Strategien der Medien“ und bedienten gleichzeitig die „Kriterien, mit denen in sozialen Netzwerken Reichweite erzielt“ werde (S. 170). Ein den Verschwörungstheorien verwandtes Phänomen stellen Lügen oder Fake News und Täuschung dar. Mit ihnen setzt sich Helmut König anhand zweier prominenter Beispiele – Wladimir Putin und Donald Trump – auseinander.^27^ Ein wichtiges Unterscheidungsmerkmal gilt es allerdings zu beachten: ‚Gute‘ Verschwörungstheorien beanspruchen oft ein gewisses Maß an Plausibilität. Trump hingegen gebe „seinen Lügen nicht einmal mehr den Anschein von Plausibilität“ (S. 205). Das kennzeichnet auch die Verschwörungstheorien, die er verbreitet: „Die Lüge über den Geburtsort von Obama, die wahnwitzige Pizzagate-Geschichte über Hillary Clinton, die Behauptung, dass führende Politiker der Demokraten sich an Satanismus-Ritualen beteiligten, – je absurder die Behauptungen […], desto besser“ (S. 215 f.).

## „Querdenker“ und Verschwörungstheorien

Eine vergleichsweise ‚junge‘ Bewegung hat sich nichtsdestotrotz in kürzester Zeit zu einem höchst wirkungsvollen Verstärker und *spreader* von Verschwörungserzählungen entwickelt: die „Querdenker“, eine im Frühjahr 2020 in Stuttgart vor dem Hintergrund des Corona-Lockdowns unter dem Namen „Querdenken 711“ ins Leben gerufene Protestbewegung. Mehrere Arbeiten haben seither dieses erstaunliche Phänomen untersucht, drei in publizierter Form vorliegende und in der öffentlichen Debatte rezipierte ‚Pionierstudien‘ werden im Folgenden vorgestellt.

Mit einem unterschiedliche Perspektiven vereinenden Sammelband^28^ möchte der ebenso produktive wie engagierte Zeithistoriker Wolfgang Benz zum „Verständnis einer politischen und sozialen Bewegung“ beitragen, die sich „in der Absage an die demokratisch verfasste Gesellschaft und den parlamentarisch legitimierten Staat zunehmend radikalisiert“ habe (S. 24). Das Spektrum der überwiegend von Geschichts- und Sozialwissenschaftler_innen verfassten Beiträge reicht von der sehr anschaulichen Schilderung von Kontakten zwischen ‚einfachen‘ Bürger_innen mit ‚ihren‘ Abgeordneten (Angelika Censebrunn-Benz) über Antisemitismus als „Bindekitt für Verdrossene und Verweigerer“ (Juliane Wetzel), „Querdenken und Corona-Leugnung als Strömung der Lebensreformbewegung“ (Andreas Speit) und den Einfluss fundamentalistischer Sekten auf die „Querdenken“-Bewegung (Recherchenetzwerk AS) bis zur Beschäftigung mit den (bislang) erfolglosen Versuchen der AfD, die „Querdenken“-Bewegung auf ihre Seite zu ziehen (Gudrun Hentges, Gerd Wiegel). Hier interessiert jedoch hauptsächlich der Zusammenhang zwischen der „Empörungsmaschine“ unter dem Label „Querdenken“, die „Zehntausende auf die Straße treibt“ (Josef-Otto Freudenreich: Aufruhr in Stuttgart, S. 113) und Verschwörungstheorien. Peter Widmann denkt darüber nach, wie die Auseinandersetzung mit Verschwörungstheorien zur Erreichung eines wichtigen Ziels politischer Bildung genutzt werden kann, nämlich Räume für „Selbstwirksamkeitserfahrungen“ zu öffnen: schließlich habe die empirische Sozialforschung gezeigt, dass der „Verschwörungsglaube für Individuen auch die Funktion“ habe, „Gefühle des Kontrollverlusts zu kompensieren“ (S. 44). Mit den Versuchen, mithilfe der Verschwörungserzählung vom „Great Reset“ Synergieeffekte zwischen „Neuer Rechter“ und Corona-Protesten zu erzielen, befasst sich der oben bereits erwähnte Volker Weiß. Und Benz gibt Antworten auf die Frage, warum Verschwörungsmythen so attraktiv erscheinen: Zum „Wesen der Verschwörungsfantasie als Botschaft“ gehöre es, dass sie „unmittelbar geglaubt und nicht kritisch reflektiert wird, weil sie Wünschen und Erwartungen entspricht“. Gemeinsames Merkmal sei das „Absurde, die Verweigerung der Gesetze der Logik, der Anspruch auf gültige Welterklärung mit Argumenten und ‚Beweisen‘, die fern jeder Rationalität“ seien und deshalb „nicht verifiziert“ werden könnten, sondern „durch Glauben gültig“ würden. Verschwörungsmythen „nutzen als Ausgangspunkt reale Begebenheiten, sie bedienen Gefühle des Bedrohtseins, der Angst, der Abwehr wirklicher oder imaginärer Gefahr, sie markieren Feinde, grenzen sie aus und schaffen Zusammenhalt unter den Gläubigen“ (S. 78). Benz verdeutlicht die Unterschiede zwischen Gerüchten und Verschwörungstheorien, weist am Beispiel des renommierten DDR-Naturwissenschaftlers Jakob Segal, der die These vom „künstlich in einem Labor“ erzeugten AIDS-Virus in Umlauf gebracht hatte, darauf hin, dass auch ‚seriöse‘ Wissenschaftler_innen und Intellektuelle oder linke Kreise nicht gegen Verschwörungstheorien gefeit seien (S. 86), und geht abschließend kurz auf die sinnstiftende Funktion und den „Unterhaltungswert“ (S. 98) von Verschwörungsmythen ein. Sein Fazit: „Legenden über geheime Mächte und deren Machenschaften bestätigen die Ohnmacht des Individuums und rufen das Kollektiv der Gläubigen zur Selbstverteidigung […] gegen konspirativ definierte Feinde auf“. Die ‚Gläubigen‘ lebten in einer „hermetischen Welt“, in der „geheime Mächte“ im Hintergrund die Strippen zögen, die politischen und gesellschaftlichen Eliten als „willfährige Gehilfen“ deren Pläne ausführten, beobachtet vom „dummen Volk“ und durchschaut von den wenigen „Wissenden“, die „alles erkannt“ hätten. Und dieses „Wissen“ um die „wahren Zusammenhänge“ mache „Konspirationsfantasien für viele so attraktiv“ (S. 99). Mit dieser Deutung gesellt sich Benz unmissverständlich zu den ‚Skeptikern‘: eine Position, die dem einschlägigen Forschungs-‚Mainstream‘ entspricht. Ob diese ‚anti-verschwörungstheoretische‘ Einstellung möglicherweise *grundsätzlich* einem ‚Verstehen‘ des Phänomens zuwiderläuft – darauf wird zurückzukommen sein.

Mit den Parallelen und Kontinuitäten zwischen den „Querdenkern“ der Gegenwart und der Lebensreformbewegung sowie den Inflationsheiligen um die Wende vom 19. zum 20. Jahrhundert und in den 1920er Jahren beschäftigt sich der Kulturwissenschaftler und Journalist Steffen Greiner.^29^ Er erinnert daran, dass Krisenzeiten „oft mit religiösen Bewegungen und messianischen Figuren“ einhergehen (S. 11). Ulrich Linse, an dessen Buch über die „Propheten“ und „Inflationsheiligen“ der 1920er Jahre^30^ Greiner teilweise anknüpft, hatte im Epilog ebenfalls auf „Religiosität“ als Merkmal dieser Strömung aufmerksam gemacht und die Vermutung geäußert, dass „kollektive Gewißheitsverluste im Gefolge krisenhafter Erschütterungen“ auch in den Industriestaaten der 1980er Jahre „eine immer noch vorhandene politische Religiosität eruptiv freisetzen können“^31^. Die Traditionslinie, die Linse zog, endete bei den sogenannten Neuen Religionen. Greiner erwähnt Stuttgart als „Brennglas der spirituell-volksfrommen Strömungen, die sich links und rechts der calvinistischen Geschäftigkeit bildeten“ (S. 109): „Bewegungen der Volksfrömmigkeit und Widerstand gegen kirchliche Obrigkeit, Mystik und Konservatismus, das ballt sich hier zusammen“ (S. 110). Geprägt werde die „Querdenken“-Bewegung freilich von Akteuren anderen Zuschnitts: von dem Softwareunternehmer Michael Ballweg, dem HNO-Arzt Bodo Schiffmann, dem Theatermacher Anselm Lenz, dem Sänger Xavier Naidoo^32^, dem – bereits erwähnten – früheren Radiomoderator Ken Jebsen und dem Kochbuchautor Attila Hildmann – auffälligerweise „ganz überwiegend Männer, obwohl Frauen in der Bewegung ähnlich präsent sind und zentrale Themen wie Spiritualität und alternative Medizin auch in links-feministischen Kreisen Relevanz besitzen“ (S. 118). Auf das Thema „Verschwörungstheorien“ geht Greiner nur am Rande ein. So erwähnt er beispielsweise, dass das QAnon-Narrativ in der „Querdenken“-Szene „massiv an Zuspruch“ gewonnen habe (S. 138). „Verschwörungstheorien, die in den 1980ern allenfalls theoretisch verbrämt hinter akademischem Vokabular der Postmoderne auftauchten, sind nun als zugängliche Erzählungen konstitutiv: Bill Gates, QAnon, die BRD GmbH“ (S. 249). Die eigentliche Stärke des Buches liegt indes in der plastischen Schilderung der ersten, heute weithin vergessenen „Querdenker“ der 1920er Jahre: August Engelhardt, Heinrich Goldberg, Gusto Gräser, Louis Haeusser, Max Schulze-Sölde und deren Anhänger_innen. Ob die religiöse Kontinuitätslinie, die Greiner zieht, tatsächlich repräsentativ ist für die Mehrheit der heutigen ‚Nachfolger‘, müssen weitere Forschungen zeigen.

Mittlerweile liegen auch erste empirische Arbeiten zum Thema „Querdenker“ vor. Den Anfang machten die Soziologen Oliver Nachtwey, Nadine Frei und Robert Schäfer mit einer als Download abrufbaren Studie.^33^ Das erste umfangreiche Buch hat der Konstanzer Zeithistoriker Sven Reichardt herausgegeben.^34^ Der Herausgeber und seine Autor_innen berufen sich auf die sozialwissenschaftliche Bewegungsforschung, merken aber an, dass noch nicht verlässlich abzuschätzen sei, wie „dauerhaft“ die „Querdenken“-Bewegung sein werde (S. 11). Weder lasse sich ein gemeinsames „politisches Programm“ noch eine gemeinsame „politische Ideologie“ identifizieren. Ihre Gemeinschaft sei vielmehr „in doppelter Hinsicht negativ definiert: einerseits durch die Ablehnung großer Teile der Infektionsschutzpolitik, andererseits durch das grundsätzliche Misstrauen gegenüber den politisch Handelnden, ihren wissenschaftlichen Ratgeber:innen und den etablierten Medien“ (S. 18). Die Anziehungskraft von Verschwörungstheorien auf dieses Protestmilieu ist deshalb leicht zu erklären, weil diese Erzählungen vom ‚Mainstream‘ abweichende Erklärungen, das heißt ‚Gegenwissen‘ anbieten: „Der Glaube, alles sei kontrollierbar, vermittelt den Anhänger:innen einerseits Sicherheit, leistet aber andererseits dem Denken in Feindbildern Vorschub“ (S. 19). Wie Sebastian Koos auf Grundlage einer standardisierten Befragung unter den Teilnehmerinnen und Teilnehmern einer Demonstration vom Oktober 2020 in Konstanz zeigen kann, ist die Zustimmung zu bestimmten, vergleichsweise ‚alltäglichen‘ Verschwörungstheorien recht hoch: Immerhin 70 beziehungsweise 75 Prozent der Befragten konnten sich vorstellen, dass einflussreiche Geschäftsleute die Bevölkerung zwangsimpfen lassen wollten und Gruppen von Wissenschaftler_innen Beweise manipulieren, erfinden oder unterdrücken, um die Öffentlichkeit zu täuschen. Allerdings werden diese hohen Zahlen dadurch etwas relativiert, dass, wie der Autor selbst einräumt, nach der *Vorstellbarkeit* der beiden Behauptungen gefragt wurde. Zustimmung zu QAnon und den in der Anhängerschaft dieser Gruppierung verbreiteten Verschwörungserzählungen äußerten hingegen nur fünf Prozent (S. 76 f.). Der Band, der noch weitere innovative und höchst informative Beiträge zu den Teilnehmer_innen der Proteste, ihrer Kommunikation im Internet sowie den rhetorischen, visuellen und interaktionalen Praktiken aus ethnografischer Sicht zu bieten hat, überzeugt vor allem durch seine breite empirische Grundlage und ist unverzichtbar für alle, die mehr über Motivation, soziale Zusammensetzung und einschlägige Netzwerke der „Querdenker“ erfahren möchten.

Nicht unerwähnt bleiben sollte an dieser Stelle schließlich eine frühe „empirische Grundlagenarbeit“ über Verschwörungstheorien auf der Basis von Fragebogen-Erhebungen zu „soziodemografischen Merkmalen“ der Anhänger_innen solcher Theorien wie Geschlecht, Alter und der Rolle der Religiosität^35^. Zu Sebastian Bartoscheks Befunden zählen insbesondere, dass „der Glaube an Verschwörungstheorien kein pathologisches Merkmal, sondern stabil in der Mitte der Gesellschaft verortet“ sei (S. 195), und dass „Geschlecht, Extremismus, Gläubigkeit, Alter und Bildungsniveau“ keinen signifikanten Einfluss auf das Interesse und die Anfälligkeit für Verschwörungstheorien hätten (S. 196). Die ziemlich disparate und unübersichtliche Präsentation der Ergebnisse schränkt den Nutzen des Buchs allerdings erheblich ein.

## Geschichtswissenschaftliche Perspektiven

Wie eingangs erwähnt, ist die Zahl neuerer einschlägiger geschichtswissenschaftlicher Arbeiten recht überschaubar. Zwar melden sich Historiker_innen gelegentlich durchaus zu Wort – einige sind in diesem Aufsatz erwähnt –, allerdings nicht mit explizit geschichtswissenschaftlichen Herangehensweisen und Methoden verpflichteten Studien. Eine Ausnahme bildet die jüngste Veröffentlichung von Richard J. Evans.^36^ Ausgehend von der Beobachtung, dass nirgendwo „die Ausbreitung von Verschwörungstheorien und ‚alternativen Fakten‘ offensichtlicher“ sei als in revisionistischen Darstellungen der Geschichte des ‚Dritten Reichs‘ (S. 11), seziert der britische Historiker auf geradezu vorbildhafte Art und Weise fünf vermeintliche Verschwörungen aus der Welt des Nationalsozialismus: Die „Protokolle der Weisen von Zion“, die Dolchstoßlegende, den Reichstagsbrand, den überraschenden Flug des „Stellvertreters des Führers“, Rudolf Heß, nach Schottland und die angebliche Flucht Hitlers aus dem Bunker. Er geht dabei der Frage nach, ob diese Verschwörungserzählungen Hitler beeinflusst hätten, ob er manche selbst angeregt oder sich sogar „aktiv an ihrer Verbreitung beteiligt“ habe (S. 18). Dezidiert – und völlig zu Recht – weist er aber die Ansicht zurück, es spiele „keine Rolle“, ob einzelne Erzählungen „wahr oder falsch“ seien, es „komme lediglich darauf an, dass sie (selbst wenn sie wie die *Protokolle* auf eindeutig gefälschten oder verfälschten Beweisen beruhten) eine grundlegende Wahrheit enthüllten und daher in einem tieferen Sinn wahr seien, als es das bloß Empirische sein könnte“. Eine solche Argumentation stelle nämlich „das Wesen der Wahrheit selbst zur Debatte“ (S. 22).

Welche Befunde präsentiert Evans? Zu den „Protokollen der Weisen von Zion“ stellt er beispielsweise fest, dass sie „eher indirekten als direkten Einfluss auf Hitler und die Nationalsozialisten“ gehabt hätten, jedenfalls seien ihre Taten nicht auf die Lektüre der „Protokolle“ zurückzuführen: „Tatsächlich waren sie für die Nationalsozialisten keine Offenbarung, sondern lediglich eine Bestätigung dessen, was sie bereits ‚wussten‘“ (S. 69). Auch zur Bedeutung der Dolchstoßlegende merkt er relativierend an, dass ihre Wirkung „auf einen kleinen, allerdings lautstarken und einflussreichen Rand des politischen Systems der Weimarer Republik“ beschränkt und von den Nationalsozialisten in ihrer Propaganda „kaum“ eingesetzt worden sei; die „Legitimität“ der Republik sei „weniger durch die Dolchstoßlegende […] als vielmehr durch ein allgemeineres Gefühl, das die Geburt der Demokratie mit der nationalen Demütigung der Friedensbedingungen und der Kriegsschuldklausel des Versailler Vertrags verknüpfte“, untergraben worden (S. 121). Im Fall des Reichstagsbrands widerspricht Evans sowohl der Lesart der Nationalsozialisten, Kommunisten hätten den Reichstag als „Auftakt einer gewaltsamen kommunistischen Revolution“ in Brand gesteckt, als auch der umgekehrten Version, die Nazis selbst seien die Brandstifter gewesen, denn die Indizien seien „überwältigend“ (S. 169). Und auch die zahlreichen Verschwörungserzählungen, die sich um Heß’ Schottlandflug ranken, verweist er, nachdem er das jeweils zugrundeliegende „Gewebe aus Erfindungen und Fälschungen“ (S. 213) ebenso akribisch wie genüsslich zerpflückt hat, ins ‚Reich der Fantasie‘: „Heß’ Mission beruhte auf seinen eignen Illusionen, nicht auf der Täuschung anderer“ (S. 222). Noch bizarrer wird es im letzten Kapitel des Buchs, das sich den Verschwörungserzählungen um Hitlers angebliche Flucht aus dem Bunker widmet. Dass „Verschwörungstheorien, die sich um Hitler drehen, heute zunehmend verbreitet und in manchen Fällen wiederbelebt werden“, so Evans Fazit, sei „Teil eines umfassenden Trends“, gebildet aus unterschiedlichen Strömungen, die darauf zielten, „die Grenze zwischen Wahrheit und Fiktion zu verwischen“ oder „alternative ‚Wahrheiten‘ zu präsentieren, von denen jede angeblich der Realität“ entspreche (S. 306.) Zu den „beunruhigendsten Aspekten einiger Verschwörungstheorien gehört der offensichtliche Glaube, dass es im Grunde keine Rolle spielt, ob sie wahr sind oder nicht“. Aber, so Evans voller Überzeugung, spiele es sehr wohl eine Rolle: „Herauszufinden, was in der Geschichte wirklich geschehen ist, ist schwierig“ und „erfordert viel harte Arbeit“, insbesondere die „genaue Untersuchung von Belegen“ und die Bereitschaft, „seine Vorurteile und vorgefassten Ansichten aufzugeben, wenn die Belege gegen sie sprechen“ (S. 307).

## Ethnografisch-kulturwissenschaftliche Ansätze: Radikale Öffnung zum ‚Gegenwissen‘

Einen entgegengesetzten Weg gehen zwei ethnografisch beziehungsweise kultursoziologisch angelegte, höchst interessante und zum Teil auch beunruhigende Arbeiten. Alan Schink, der als Lehrbeauftragter für Qualitative Forschungsmethoden an der Paracelsus Medizinischen Privatuniversität Salzburg und als „Achtsamkeits- und Stressreduktionstrainer“ arbeitet, legt gleich zu Beginn seiner Dissertation^37^ die Prämissen seines methodischen Zugriffs in apodiktischer Manier offen: „um das Verschwörungsdenken wirklich zu verstehen“, müsse man sich „einer kognitiven Dissonanz aussetzen“, die „gehegte Komfortzone verlassen und sich radikal für eine Wirklichkeit öffnen, die die eigene Wahrnehmungsmatrix in ihren Grundfesten irritiert“. Erst wenn man „dieses Denken so, von ‚innen‘ her, durchlebt, seine ‚schmutzigen‘ Untergründe für eine Zeitlang durchwatet, die Wahrheit gesucht“ habe und „in diverse Fallen getappt“ sei, könne man „zurückkehren zum ‚Business as usual‘, zur distanzierten Beschreibung und Einordnung des Verschwörungsdenkens in den gesellschaftlich-kulturwissenschaftlichen Kontext“ (S. 1). Dermaßen eingestimmt, erwartet die Leser_innen eine Reise in eine Welt, die gekennzeichnet ist von „Misstrauen gegenüber dem Offenbaren, Offensichtlichen oder Offiziellen“ (S. 7). Das Verschwörungsdenken „vermutet hinter der ‚Oberfläche‘ eine verborgene oder unterdrückte Realität“, und weil sich dieses „Verborgene“ der Beobachterin oder dem Beobachter „in seiner Gänze“ entziehe und nur „in Spuren“ zeige, sei das Verschwörungsdenken „durch eine *Spannung* und Erregung geprägt, die sich, je nach Kontext, in *Neugier* oder *Begierde, Furcht, Besorgnis, Zorn* oder *Wut* ausdrücken [Hervorh. im Orig.]“ könne (S. 8). Schink begreift „‚Verschwörungstheorien‘ gerade deswegen als ‚heterodox‘“, weil diese Deutungsmuster – und hier zitiert er Pierre Bourdieu – „‚die offizielle Weise, die Welt zu denken‘ und von ihr innerhalb einer vertrauten und ‚rationalen‘ Kommunikationsgemeinschaft ‚zu sprechen‘, hinterfragen und diskreditieren“ (S. 11).

Der inhaltlich-empirische Teil der Studie beginnt mit einer „autoethnographischen Selbstverortung“ des Autors: anhand von „Bildzeugnissen, Tagebuchfragmenten und Erinnerungsrekonstruktionen wird das Doppelleben und werden die verschiedenen Lebensabschnitte und damit verbundenen Identitäten des autobiographischen Subjekts als Maskierungen eines Fremden, Verschwörungstheoretikers, spirituell Suchenden, Ethnographen […] introspektiv verarbeitet“ (S. 12). Die Leser_innen können miterleben, wie Schink „immer tiefer […] in die Welt der Verschwörungen“ (S. 72) hineingeriet. Im nächsten Kapitel liegt der Fokus auf der „Begriffsbildung, wobei bereits soziale Praktiken und Dynamiken der Diskreditierung, Stigmatisierung und Tabuisierung des Verschwörungswissens mitthematisiert werden“ mit dem Ziel, „das Verschwörungsdenken der modernen Konspirationskultur analytisch zu durchdringen“ (S. 85). Mit dem Begriff der „Konspirationskultur“ möchte Schink verdeutlichen, dass „das Konspirative tief in unsere kulturelle Matrix eingeschrieben“ sei (S. 154). Das umfangreichste Kapitel handelt unter der Überschrift „Verschwörungen im Deutungskonflikt“ von verschiedenen realen und ‚eingebildeten‘ Verschwörungen von „9/11“ über angebliche Medienkonspirationen und „False-Flag-Operationen“ bis zu anti-verschwörungstheoretischen Diskursen, Praktiken und Plattformen wie den „Skeptikern“, der Praxis des „Debunking“ oder Wikipedia als „Watchblog“. Im nächsten Kapitel stellt Schink einige wichtige Akteure und Bewegungen der ‚Gegenöffentlichkeit‘ vor, beispielsweise das „9/11 Truth Movement“ oder „KenFM“, eines der einflussreichsten und „klickstärksten“ Portale (S. 356). Abschließend wird der „okkulte Bereich der Konspirationskultur schlaglichtartig“ beleuchtet (S. 395).

Das Buch hinterlässt beim Rezensenten einen zwiespältigen Eindruck: Auf der einen Seite bietet es präzise und freimütige Einblicke in das Milieu und das Denken von Verschwörungstheoretikern. So weckt der Autor beispielsweise Verständnis für gewisse charakteristische Einstellungen wie der „Faszination bei der Suche nach Spuren oder dem ‚Rätsel-lösen‘“, der erfolgreichen „Diskreditierung einer ‚Fassade‘“ oder der „Enthüllung“ eines Komplotts (S. 86). Auch der wiederholte Verweis auf tatsächliche Verschwörungen lässt es geraten erscheinen, pauschale, vorschnelle Urteile über entsprechende Theorien zu vermeiden. Und der „Leitgedanke“ der Studie Schinks, dass es „Kontexte und Umstände“ gebe, „in denen das Verschwörungsdenken vernünftig – ‚paranoia within reason‘ – ist“ (S. 127), erscheint durchaus bedenkenswert. Auf der anderen Seite irritiert die oft recht unkritisch wirkende Parteinahme für Verschwörungstheoretiker wie Mathias Bröckers, Daniele Ganser und Ken Jebsen, mit dessen Kanal Schink sich nach eigener Aussage in einer bestimmten Phase „mehr und mehr“ identifizierte (S. 360); selbst Alex Jones, amerikanischer ‚Star‘ der Szene und geschäftstüchtiger *spreader* auch der abstrusesten und politisch gefährlichen Erzählungen, wird bedauert, weil er durch die Sperrung auf YouTube und Twitter angeblich „seiner beruflichen Existenzgrundlage beraubt“ werde (S. 318). Dass Schink selbst von seiner erfolgreichen „Distanzierung“ von diesem „Feld“ vor allem „durch eine Distanzierung von entsprechenden digitalen Praktiken und mit diesen zusammenhängenden Emotionsmustern und Wissensbeständen“ (S. 430) sowie durch „die Entdeckung der Liebe und des Vertrauens in einer Beziehung“ (S. 431) berichtet, zeigt zumindest, dass ein Ausstieg aus diesem Milieu unter bestimmten Bedingungen möglich ist.

Wie Schink bezieht auch der Soziologe Jaron Harambam eine agnostische Haltung gegenüber dem Wahrheitswert von Verschwörungstheorien und taucht in den Alltag von Menschen ein, die im niederländischen Verschwörungsmilieu aktiv sind, um besser verstehen zu können, was heutzutage die Attraktivität solcher Theorien ausmacht.^38^ Nach einer der Klärung der Begriffe und der Erläuterung der Relevanz des Themas dienenden Einleitung und einem Kapitel zur Methodologie und zur Quellenbasis – insbesondere einschlägige Webseiten, Organisationen, Videos und Publikationen sowie Interviews mit 21 Personen unterschiedlichen Alters, Geschlechts und unterschiedlicher sozialer Herkunft – setzt sich Harambam mit den wichtigsten in den Niederlanden kursierenden Erzählungen auseinander, die, wie hierzulande auch, um die Macht einzelner Unternehmer, großer Konzerne, einflussreicher Medien und Wissenschaftler kreisen. Besonders informativ und aufschlussreich ist das vierte Kapitel, das auf der Teilnahme an einer Veranstaltung des „conspiracy rock star[s]“ (S. 107), David Icke, in London basiert und den ‚Erweckungscharakter‘ der Show betont. Harambams Fazit: „David Icke brings the heavens and Earth together in one extraordinary master narrative of banking scams, multidimensional universes, reptilian races, and institutional forms of mind control“ (S. 125). Anschließend fragt er nach den Gründen für die Hinwendung zu Verschwörungstheorien, die er nicht zuletzt im Zusammentreffen privater und öffentlicher Probleme sieht. Im vorletzten Kapitel wendet er sich gegen die Stigmatisierung der Anhänger_innen von Verschwörungstheorien, im letzten erläutert er, warum und wie Verschwörungsgläubige die epistemische Autorität anfechten. Zusammenfassend bekräftigt Harambam seinen Zugriff, „to understand instead of pathologize the contemporary prevalence of conspiracy theories. My goal was not to condemn or discard them, but to grasp the meaning that these alternative forms of knowledge have for the people involved with them“ (S. 206). Er lobt die grundsätzliche Skepsis gegenüber den Realitäten des Alltags und erkennt an, dass „conspiracy theorists […] set out in search of hidden or latent meanings, asking if there are any clues or symbols pointing to the *real truth* [Hervorh. im Orig.]“ (S. 214). Immerhin beschließt Harambam seine Studie mit einem vergleichsweise vagen Bekenntnis: „While I may side with conspiracy theorists on *procedural* terms, I do not (necessarily) side with them on *substantial* terms [Hervorh. im Orig.]“ (S. 238).

## Wie mit Verschwörungserzählungen umgehen?

Schlösse man sich den neutral-verständnisvollen Standpunkten Schinks und Harambams an, wären Überlegungen zum Umgang mit Verschwörungstheorien und deren Anhänger_innen völlig überflüssig. Tatsächlich versorgen einige neuere Veröffentlichungen ihre Leser_innen mit entsprechenden Ratschlägen – weil die Autor_innen solche Erzählungen offensichtlich für gefährlich und dem Zusammenleben in einer Demokratie abträglich halten. Nocun und Lamberty^39^ erwähnen ein „Fünf-Phasen-Modell des prosozialen Verhaltens“: „Wahrnehmung der Situation“, „Interpretation der Situation“, „Verantwortungsübernahme“, „Einschätzung der eigenen Fähigkeiten“, „Handeln“ (S. 277–279). Eine „allgemein gültige Empfehlung“ zum Umgang mit Verschwörungsgläubigen gebe es allerdings nicht, weil „die Forschung in diesem Bereich noch in den Kinderschuhen“ stecke und die Fälle „sehr unterschiedlich“ seien (S. 280). Generell empfehlen die Autorinnen, möglichst früh zu intervenieren. Angehörige, die das Gefühl haben, „der Betroffene würde ihnen nach und nach entgleiten“ (S. 294), sollten auch nicht zögern, eventuell professionelle Hilfe in Anspruch zu nehmen. Auch die Journalistin Franzi von Kempis^40^ hat einige praktische Ratschläge parat. Sie hält Widerspruch gerade bei „antidemokratischen, antisemitischen Inhalten“ für wichtig, schon um die „schweigende Mehrheit“ nicht indirekt zu bestärken. Auch rät sie, das Weltbild des Gegenübers nicht frontal infrage zu stellen, falsche Informationen beim Widerlegen nicht zu wiederholen, sich auf wenige konkrete Argumente zu konzentrieren und vor allem „offen“ zu bleiben für abweichende Ansichten zu bestimmten politischen, wirtschaftlichen und gesellschaftlichen Ereignissen und Prozessen – zumindest, solange verschwörungstheoretische Deutungen „trotz Gegenbeweisen“ nicht fallengelassen werden (S. 107). Und Michael Blume, Beauftragter der Landesregierung Baden-Württemberg gegen Antisemitismus, erinnert daran, dass es weniger um ein rationales als vielmehr um ein emotionales Thema gehe^41^: Deshalb sei es nicht sinnvoll, mit Verschwörungsgläubigen über deren ‚Theorien‘ zu diskutieren, sondern vielmehr „gezielt die Emotionen und die Ängste, die sich hinter der Anfälligkeit für Verschwörungsmythen verbergen“ (S. 134), zu erfragen. Im Gegensatz zu diesen Autor_innen glauben Anton und Schink,^42^ dass „der Kampf gegen Verschwörungstheorien sich nicht nur gegen tatsächlich problematische oder ‚gefährliche‘ – etwa irreführende, hasserfüllte, antisemitische oder rassistische – Inhalte richtet, sondern selbst problematisch oder gar bedrohlich für demokratische Diskurse werden kann, indem möglicherweise relevante und unterrepräsentierte Stimmen ausgeschlossen oder stigmatisiert werden“ (S. 264). Konkrete Belege für diese Sorge bleiben sie allerdings schuldig.

## Streit um Wahrheit

Die ‚Wahrheit‘ steht bei Kritikern wie Anhängerinnen und Anhängern von Verschwörungserzählungen gleichermaßen hoch im Kurs. Der oben erwähnte Historiker Richard Evans beispielweise ist überzeugt: „Es gibt nur eine Wahrheit, auch wenn sie manchmal schwer zu finden ist“ (S. 307). Der Philosoph und Schriftsteller Jan Skudlarek konstatiert^43^: „Wahre Verschwörungstheorien gibt es nicht“ (S. 196) und „gefühlte“ Wahrheiten auch nicht (S. 203). Andererseits warb die AfD im letzten Bundestagswahlkampf mit dem Slogan „Mut zur Wahrheit“ um Wählerinnen und Wähler. Auch Verschwörungserzählungen beanspruchen, ‚wahr‘ zu sein.

Wie die Basler Literaturwissenschaftlerin Nicola Gess in einem schmalen, aber klugen Buch in exemplarischer Absicht nachweist^44^, sind für populistisches und verschwörungstheoretisches Denken allerdings hauptsächlich „Halbwahrheiten“ kennzeichnend. Halbwahrheiten sind, so Gess, „Äußerungen, die nur zu einem Teil auf tatsächlichen Ereignissen, zu einem anderen aber auf fiktiven Inhalten basieren; Äußerungen, die reale Sachverhalte übertreiben, umdeuten oder in falsche Zusammenhänge stellen; oder auch Äußerungen, die wesentliche Informationen weglassen“ (S. 8). Der Begriff der Halbwahrheit impliziere „sowohl das Festhalten an als auch die Diffusion einer klaren Unterscheidung von ‚wahr‘ und ‚falsch‘“. Halbwahrheiten operierten nämlich nicht „nach dem binären Code *wahr/falsch*, sondern *glaubwürdig/unglaubwürdig *[Hervorh. im Orig.]“ (S. 13). Am Beispiel eines von Ken Jebsen geposteten Videos mit dem Titel „Bill Gates kapert Deutschland“, das „innerhalb von nur drei Tagen mehr als 5 Millionen Mal auf verschiedenen Plattformen“ aufgerufen wurde und das dessen Version der angeblichen Corona-Verschwörung präsentiert, demonstriert Gess, welche Rolle Halbwahrheiten für Jebsens ‚Beweisführung‘ spielen. Alles in allem plädiert sie für einen „differenzierten Wahrheitsbegriff“ als Mittel gegen einen „zynischen Relativismus“, der „autoritäre Züge annehmen“ könne (S. 100).

Anton und Schink fordern am Ende ihres Buches über den „Kampf um die Wahrheit“ zu einem toleranten, verständigungsbereiten, differenzierten und der ‚Freiheit‘ verpflichteten Umgang mit Verschwörungstheorien auf. Das klingt zunächst einmal gut und beherzigenswert, läuft am Ende aber auf eine Nivellierung unterschiedlichster Wissensbestände hinaus. Ist es wirklich ratsam, mit Anhänger_innen der Erzählung von der „jüdischen Weltverschwörung“ tolerant umzugehen? Soll man sich gegenüber QAnon-Gläubigen tatsächlich verständigungsbereit zeigen? Der Anspruch, ‚nach der Wahrheit zu streben‘, sollte nicht den „Querdenkern“ und Verschwörungstheoretikern überlassen werden: „Denn wenn es keine allgemein gültige Wahrheit mehr gibt, auf die sich alle einigen können, dann gibt es auch keine Möglichkeit mehr, gemeinsam zu handeln.“^45^ Und das wäre für den Fortbestand der Demokratie höchst bedrohlich.

## Besprochene Literatur

- Anton, Andreas/Schink, Alan: Der Kampf um die Wahrheit. Verschwörungstheorien zwischen Fake, Fiktion und Fakten, 336 S., Komplett Media, München 2021.
- Bartoschek, Sebastian: Bekanntheit von und Zustimmung zu Verschwörungstheorien. Eine empirische Grundlagenarbeit, 356 S., JMB, Hannover ^5^2020. Zugl.: Münster (Westfalen), Univ., Diss., 2013.
- Benz, Wolfgang (Hrsg.): Querdenken. Protestbewegung zwischen Demokratieverachtung, Hass und Aufruhr, 318 S., Metropol, Berlin 2021.
- Blume, Michael: Verschwörungsmythen. Woher sie kommen, was sie anrichten, wie wir ihnen begegnen können, 160 S., Patmos, Ostfildern 2020.
- Bodner, John, u. a.: Covid-19 Conspiracy Theories. QAnon, 5G, the New World Order and other Viral Ideas, 263 S., McFarland, Jefferson, N.C. 2021.
- Butter, Michael: „Nichts ist, wie es scheint“. Über Verschwörungstheorien, 271 S., Suhrkamp, Berlin 2018.
- Butter, Michael/Knight, Peter (Hrsg.): Routledge Handbook of Conspiracy Theories, 700 S., Routledge, London/New York 2021.
- Evans, Richard J: Das Dritte Reich und seine Verschwörungstheorien. Wer sie in die Welt gesetzt hat und wem sie nutzen – Von den „Protokollen der Weisen von Zion“ bis zu Hitlers Flucht aus dem Bunker, übers. v. Klaus-Dieter Schmidt, 368 S., DVA, München 2021 (engl. 2020).
- Fielitz, Maik/Marcks, Holger: Digitaler Faschismus. Die sozialen Medien als Motor des Rechtsextremismus, 256 S., Duden, Berlin 2020.
- Frizzoni, Brigitte (Hrsg.): Verschwörungserzählungen, 356 S., Königshausen & Neumann, Würzburg 2020.
- Gess, Nicola: Halbwahrheiten. Zur Manipulation von Wirklichkeit, 157 S., Matthes & Seitz, Berlin 2021.
- Götz-Votteler, Katrin/Hespers, Simone: Alternative Wirklichkeiten? Wie Fake News und Verschwörungstheorien funktionieren und warum sie Aktualität haben, 214 S., transcript, Bielefeld 2019.
- Greiner, Steffen: Die Diktatur der Wahrheit. Eine Zeitreise zu den ersten Querdenkern, 272 S., Klett-Cotta, Stuttgart 2022.
- Harambam, Jaron: Contemporary Conspiracy Culture. Truth and Knowledge in an Era of Epistemic Instability, 256 S., Routledge, London/New York 2020.
- Harder, Bernd: Verschwörungstheorien. Ursachen – Gefahren – Strategien, 166 S., Alibri, Aschaffenburg 2018.
- Kempis, Franzi von: Anleitung zum Widerspruch. Klare Antworten auf populistische Parolen, Vorurteile und Verschwörungstheorien, 288 S., Mosaik, München 2019.
- Kleffner, Heike/Meisner, Matthias (Hrsg.): Fehlender Mindestabstand. Die Coronakrise und die Netzwerke der Demokratiefeinde, 352 S., Herder, Freiburg i. Br. 2021.
- Knight, Peter: Conspiracy Culture. From the Kennedy Assassination to the X Files, 300 S., Routledge, London 2000.
- König, Helmut: Lüge und Täuschung in den Zeiten von Putin, Trump & Co., 360 S., trancript, Bielefeld 2020.
- Konda, Thomas Milan: Conspiracies of Conspiracies. How Delusions Have Overrun America, 432 S., Chicago UP, Chicago, IL 2019.
- Nachtwey, Oliver/Frei, Nadine/Schäfer, Robert: Politische Soziologie der Corona-Proteste. Grundauswertung, Universität Basel, 17. Dezember 2020, URL: 10.31235/osf.io/zyp3f [Zugriff: 09.05.2022].
- Nocun, Katharina/Lamberty, Pia: Fake Facts. Wie Verschwörungstheorien unser Denken bestimmen, 352 S., Quadriga, Köln 2020.
- Nyder, C. E.: Great Reset. Der Angriff auf Demokratie, Nationalstaat und bürgerliche Gesellschaft, 384 S., Kopp, Rottenburg 2021.
- Önnerfors, Andreas/Krouwel, André (Hrsg.): Europe. Continent of Conspiracies. Conspiracy Theories in and about Europe, 282 S., Routledge, London/New York 2021.
- Orzechowski, Peter: Durch Corona in die Neue Weltordnung, 221 S., Kopp, Rottenburg 2021.
- Osrainik, Flo: Das Corona Dossier. Unter falscher Flagge gegen Freiheit, Menschenrechte und Demokratie, 512 S., Rubikon, Mainz ^2^2021.
- Pinchbeck, Daniel: Conspiranoia. Die verratenen Staaten von Amerika, 128 S., Europa, München/Wien 2021.
- Reichardt, Sven (Hrsg.): Die Misstrauensgemeinschaft der „Querdenker“. Die Corona-Proteste aus kultur- und sozialwissenschaftlicher Perspektive, 323 S., Campus, Frankfurt a. M. 2021.
- Reinalter, Helmut (Hrsg.): Handbuch der Verschwörungstheorien, 345 S., Salier, Leipzig 2018.
- Schink, Alan: Verschwörungstheorie und Konspiration. Ethnographische Untersuchungen zur Konspirationskultur, 526 S., Springer, Wiesbaden 2020.
- Schönberger, Christoph und Sophie (Hrsg.): Die Reichsbürger. Verfassungsfeinde zwischen Staatsverweigerung und Verschwörungstheorie, 203 S., Campus, Frankfurt a. M. 2020.
- Schreyer, Paul: Chronik einer angekündigten Krise. Wie ein Virus die Welt verändern konnte, 176 S., Westend, Frankfurt a. M. 2020.
- Schumacher, Gerhard: Vorwärts in die Vergangenheit. Durchblick durch einige „reichsbürgerliche“ Nebelwände, 461 S., JMB, Hannover ^2^2018.
- Skudlarek, Jan: Wahrheit und Verschwörung. Wie wir erkennen, was echt und wirklich ist, 217 S., durchges. und erg. Ausgabe, Reclam, Ditzingen 2021.
- Stumpf, Sören/Römer, David (Hrsg.): Verschwörungstheorien im Diskurs. Interdisziplinäre Zugänge (Zeitschrift für Diskursforschung, 4. Beiheft), 336 S., Beltz, Weinheim 2020.
- Wippermann, Wolfgang: Verschwörungen. Von Catilina bis al-Kaida, 183 S., Metropol, Berlin 2018.

## Footnotes

1 Wippermann, Wolfgang: Verschwörungen. Von Catilina bis al-Kaida, Metropol, Berlin 2018.

2 Anton, Andreas/Schetsche, Michael: Vielfältige Wirklichkeiten. Wissenssoziologische Überlegungen zu Verschwörungstheorien, in: Stumpf, Sören/Römer, David (Hrsg.): Verschwörungstheorien im Diskurs. Interdisziplinäre Zugänge (Zeitschrift für Diskursforschung, 4. Beiheft), Beltz, Weinheim 2020, S. 88–115, hier S. 88.

3 Einen gegenüber Verschwörungstheorien im Kontext der COVID-19-Pandemie kritischen Überblick bieten Bodner, John u. a.: Covid-19 Conspiracy Theories. QAnon, 5G, the New World Order and other Viral Ideas, McFarland, Jefferson, N.C. 2021.

4 Vgl. beispielsweise Nocun, Katharina/Lamberty, Pia: Fake Facts. Wie Verschwörungstheorien unser Denken bestimmen, Quadriga, Köln 2020, S. 21 (die den Begriff paradoxerweise im Untertitel dennoch verwenden); und Bratich, Jack Z.: Conspiracy Panics. Political Rationality and Popular Culture, SUNY Press, Albany, NY 2008, S. 4.

5 Vgl. Butter, Michael: „Nichts ist, wie es scheint“. Über Verschwörungstheorien, Suhrkamp, Berlin 2018 (Zitat S. 53); Hepfer, Karl: Verschwörungstheorien. Eine philosophische Kritik der Unvernunft, transcript, Bielefeld 2015, bes. S. 23–25; Anton, Andreas/Schink, Alan: Der Kampf um die Wahrheit. Verschwörungstheorien zwischen Fake, Fiktion und Fakten, Komplett Media, München 2021, bes. S. 18–20.

6 Schreyer, Paul: Chronik einer angekündigten Krise. Wie ein Virus die Welt verändern konnte, Westend, Frankfurt a. M. 2020.

7 Linden, Markus: Die Legende vom „Konformitätsdruck“. Zur zweifelhalten Kritik an der Corona-Debatte, in: Merkur 75 (2021), H. 866, S. 34–45, hier S. 39.

8 Pinchbeck, Daniel: Conspiranoia. Die verratenen Staaten von Amerika, Europa, München/Wien 2021.

9 Osrainik, Flo: Das Corona Dossier. Unter falscher Flagge gegen Freiheit, Menschenrechte und Demokratie, Rubikon, Mainz ^2^2021.

10 Blazekovic, Aurelie von: Reitschuster ausgeschlossen, in: „Süddeutsche Zeitung“, 11. März 2022.

11 Wes Geistes Kind diese „kritischen Polizistinnen und Polizisten“ sind, ist in einer an Perfidie, Häme und Dummheit – „Emanuel [sic!] Kant“ – schwer zu überbietenden „Pressemitteilung“ zum Krieg Russlands gegen die Ukraine unter der Überschrift „Warum kapituliert die Ukraine nicht?“ nachzulesen, URL: <https://www.kritische-polizisten.de/wp-content/uploads/2022/03/Hurra-Patriotismus-02.03.2022.pdf> [Zugriff: 14.03.2022].

12 Nyder, C. E.: Great Reset. Der Angriff auf Demokratie, Nationalstaat und bürgerliche Gesellschaft, Kopp, Rottenburg 2021.

13 Orzechowski, Peter: Durch Corona in die Neue Weltordnung, Kopp, Rottenburg 2021.

14 Reinalter, Helmut (Hrsg.): Handbuch der Verschwörungstheorien, Salier, Leipzig 2018.

15 Butter, Michael/Knight, Peter (Hrsg.): Routledge Handbook of Conspiracy Theories, Routledge, London/New York 2021.

16 Butter: „Nichts ist, wie es scheint“ (wie Anm. 5); Knight, Peter: Conspiracy Culture. From the Kennedy Assassination to the X Files, Routledge, London 2000.

17 Önnerfors, Andreas/Krouwel, André (Hrsg.): Europe. Continent of Conspiracies. Conspiracy Theories in and about Europe, Routledge, London/New York 2021.

18 Konda, Thomas Milan: Conspiracies of Conspiracies. How Delusions Have Overrun America, Chicago UP, Chicago, IL 2019.

19 Frizzoni, Brigitte (Hrsg.): Verschwörungserzählungen, Königshausen & Neumann, Würzburg 2020.

20 Schönberger, Christoph und Sophie (Hrsg.): Die Reichsbürger. Verfassungsfeinde zwischen Staatsverweigerung und Verschwörungstheorie, Campus, Frankfurt a. M. 2020.

21 Schumacher, Gerhard: Vorwärts in die Vergangenheit. Durchblick durch einige „reichsbürgerliche“ Nebelwände, JMB, Hannover ^2^2018.

22 Fielitz, Maik/Marcks, Holger: Digitaler Faschismus. Die sozialen Medien als Motor des Rechtsextremismus, Duden, Berlin 2020.

23 Anton/Schink: Kampf (wie Anm. 5).

24 Camus, Renaud: Le grand remplacement, David Reinharc, Neuilly-sur-Seine 2011; eine „Zusammenstellung und Übersetzung“ in deutscher Sprache besorgte Martin Lichtmesz, eine der zentralen Figuren der „identitären Bewegung“: Revolte gegen den Großen Austausch, Antaios, Schnellroda 2016.

25 Kleffner, Heike/Meisner, Matthias (Hrsg.): Fehlender Mindestabstand. Die Coronakrise und die Netzwerke der Demokratiefeinde, Herder, Freiburg i. Br. 2021.

26 Götz-Votteler, Katrin/Hespers, Simone: Alternative Wirklichkeiten? Wie Fake News und Verschwörungstheorien funktionieren und warum sie Aktualität haben, transcript, Bielefeld 2019.

27 König, Helmut: Lüge und Täuschung in den Zeiten von Putin, Trump & Co., transcript, Bielefeld 2020.

28 Benz, Wolfgang (Hrsg.): Querdenken. Protestbewegung zwischen Demokratieverachtung, Hass und Aufruhr, Metropol, Berlin 2021.

29 Greiner, Steffen: Die Diktatur der Wahrheit. Eine Zeitreise zu den ersten Querdenkern, Klett-Cotta, Stuttgart 2022.

30 Linse, Ulrich: Barfüßige Propheten. Erlöser der zwanziger Jahre, Siedler, Berlin 1983.

31 Ebd., S. 237.

32 Naidoo hat sich im April 2022 öffentlich von verschwörungstheoretischen „Irrwegen“ distanziert. Wie dauerhaft diese Distanzierung ist, bleibt abzuwarten.

33 Nachtwey, Oliver/Frei, Nadine/Schäfer, Robert: Politische Soziologie der Corona-Proteste. Grundauswertung, Universität Basel, 17. Dezember 2020, URL: <10.31235/osf.io/zyp3f> [Zugriff: 09.05.2022]; vgl. auch dies.: Generalverdacht und Kritik als Selbstzweck. Empirische Befunde zu den Corona-Protesten, in: Benz (Hrsg.): Querdenken (wie Anm. 28), S. 194–213.

34 Reichardt, Sven (Hrsg.): Die Misstrauensgemeinschaft der „Querdenker“. Die Corona-Proteste aus kultur- und sozialwissenschaftlicher Perspektive, Campus, Frankfurt a. M. 2021; zuvor schon Pantenburg, Johannes/Reichardt, Sven/Sepp, Benedikt: Corona-Proteste und das (Gegen‑)Wissen sozialer Bewegungen, in: Aus Politik und Zeitgeschichte 70 (2021), Nr. 3–4, S. 22–27.

35 Bartoschek, Sebastian: Bekanntheit von und Zustimmung zu Verschwörungstheorien. Eine empirische Grundlagenarbeit, JMB, Hannover ^5^2020. Zugl.: Münster (Westfalen), Univ., Diss., 2013.

36 Evans, Richard J.: Das Dritte Reich und seine Verschwörungstheorien. Wer sie in die Welt gesetzt hat und wem sie nutzen – Von den „Protokollen der Weisen von Zion“ bis zu Hitlers Flucht aus dem Bunker, übers. v. Klaus-Dieter Schmidt, DVA, München 2021 (engl. 2020).

37 Schink, Alan: Verschwörungstheorie und Konspiration. Ethnographische Untersuchungen zur Konspirationskultur, Springer, Wiesbaden 2020.

38 Harambam, Jaron: Contemporary Conspiracy Culture. Truth and Knowledge in an Era of Epistemic Instability, Routledge, London/New York 2020.

39 Nocun/Lamberty: Fake Facts (wie Anm. 4).

40 Kempis, Franzi von: Anleitung zum Widerspruch. Klare Antworten auf populistische Parolen, Vorurteile und Verschwörungstheorien, Mosaik, München 2019.

41 Blume, Michael: Verschwörungsmythen. Woher sie kommen, was sie anrichten, wie wir ihnen begegnen können, Patmos, Ostfildern 2020.

42 Anton/Schink: Kampf (wie Anm. 5).

43 Skudlarek, Jan: Wahrheit und Verschwörung. Wie wir erkennen, was echt und wirklich ist, durchges. und erg. Ausgabe, Reclam, Ditzingen 2021.

44 Gess, Nicola: Halbwahrheiten. Zur Manipulation von Wirklichkeit, Matthes & Seitz, Berlin 2021.

45 Harder, Bernd: Verschwörungstheorien. Ursachen – Gefahren – Strategien, Alibri, Aschaffenburg 2018, S. 128.

